# Resequencing of global Tartary buckwheat accessions reveals multiple domestication events and key loci associated with agronomic traits

**DOI:** 10.1186/s13059-020-02217-7

**Published:** 2021-01-12

**Authors:** Kaixuan Zhang, Ming He, Yu Fan, Hui Zhao, Bin Gao, Keli Yang, Faliang Li, Yu Tang, Qiang Gao, Tao Lin, Muriel Quinet, Dagmar Janovská, Vladimir Meglič, Jacek Kwiatkowski, Olga Romanova, Nikhil Chrungoo, Tatsuro Suzuki, Zlata Luthar, Mateja Germ, Sun-Hee Woo, Milen I. Georgiev, Meiliang Zhou

**Affiliations:** 1 Institute of Crop Sciences, Chinese Academy of Agricultural Sciences, Room 107, Ziyuan North Building, Xueyuan South Road No. 80, Haidian District, Beijing, 100081 China; 2 Research Station of Alpine Crop, Xichang Institute of Agricultural Sciences, Liangshan, 616150 Sichuan China; 3 BGI Genomics, BGI-Shenzhen, Shenzhen, 58083 Guangdong China; 4 College of Horticulture, China Agricultural University, Beijing, 100083 China; 5 Groupe de Recherche en Physiologie Végétale (GRPV), Earth and Life Institute-Agronomy (ELI-A), Université catholique de Louvain, Croix du Sud 45, boîte L7.07.13, B-1348 Louvain-la-Neuve, Belgium; 6 Gene Bank, Crop Research Institute, Drnovská 507, Prague 6, Czech Republic; 7 Agricultural Institute of Slovenia, Hacquetova ulica, Ljubljana, Slovenia; 8 Department of Plant Breeding and Seed Production, University of Warmia and Mazury in Olsztyn, Plac Łódzki 3, 10-724 Olsztyn, Poland; 9 N.I. Vavilov All-Russian Institute of Plant Genetic Resources (VIR), Bol’shaya Morskaya, 42-44, St. Petersburg, Russia 190000; 10 Department of Botany, North Eastern Hill University, Shillong, 793022 India; 11 Kyushu Okinawa Agricultural Research Center, National Agriculture and Food Research Organization, Suya 2421, Koshi, Kumamoto 861-1192 Japan; 12 Biotechnical Faculty, University of Ljubljana, Jamnikarjeva 101, SI-1000 Ljubljana, Slovenia; 13 Department of Crop Science, Chungbuk National University, Cheong-ju, Republic of Korea; 14 Group of Plant Cell Biotechnology and Metabolomics, The Stephan Angeloff Institute of Microbiology, Bulgarian Academy of Sciences, Plovdiv, Bulgaria; 15 Center of Plant Systems Biology and Biotechnology, Plovdiv, Bulgaria

**Keywords:** Buckwheat, Genomic variation, Domestication, GWAS

## Abstract

**Background:**

Tartary buckwheat (*Fagopyrum tataricum*) is a nutritionally balanced and flavonoid-rich crop plant that has been in cultivation for 4000 years and is now grown globally. Despite its nutraceutical and agricultural value, the characterization of its genetics and its domestication history is limited.

**Results:**

Here, we report a comprehensive database of Tartary buckwheat genomic variation based on whole-genome resequencing of 510 germplasms. Our analysis suggests that two independent domestication events occurred in southwestern and northern China, resulting in diverse characteristics of modern Tartary buckwheat varieties. Genome-wide association studies for important agricultural traits identify several candidate genes, including *FtUFGT3* and *FtAP2YT1* that significantly correlate with flavonoid accumulation and grain weight, respectively.

**Conclusions:**

We describe the domestication history of Tartary buckwheat and provide a detailed resource of genomic variation to allow for genomic-assisted breeding in the improvement of elite cultivars.

**Supplementary Information:**

The online version contains supplementary material available at 10.1186/s13059-020-02217-7.

## Background

Buckwheat provides balanced essential amino acids, resistance starch, vitamins, and minerals to human diets, and processes rich bioactive flavonoids, such as rutin, quercetin, (iso) vitexin, and epicatechin, with therapeutic effects on diabetes, hypertension, and hyperlipidemia [1, 2]. This gluten-free crop has been traditionally used as a staple food for centuries in many high-altitude areas, such as southwestern China, Nepal, and Bhutan, and is an important raw material for functional food production. The cultivation of buckwheat can be traced back to about 4000 years ago [3], while it is still at the early stage of domestication and exhibits severe seed shattering and plant lodging. Buckwheat belongs to the Polygonaceae family and *Fagopyrum* genus that contains 21 species. The most widely cultivated species include common buckwheat (*Fagopyrum esculentum*) and Tartary buckwheat (*Fagopyrum tataricum*), which are grown on all continents as a result of their high ecological adaptability, short growth period, and tolerance of low-nutrient conditions. The improvement of this underutilized pseudocereal crop for ideal varieties with higher yield and nutrition could be harnessed in attempts to face the booming challenges of limited land and human nutritional needs.

Tartary buckwheat (*F. tataricum*) is homostylous and self-pollinated and has higher yield and flavonoids than the self-incompatible common buckwheat [4]. It is particularly rich in rutin that has therapeutic potential in Alzheimer’s disease [5, 6]. The cultivated Tartary buckwheat has been considered to originate and domesticate in the southwestern China based on the discovery and characterization of its wild relatives, literature evidence, and traditional catering culture [7–9]. The human selection and geographical isolation have resulted in the diversity of phenotypes and genotypes, providing abundant resources for Tartary buckwheat breeding. However, current cultivars of Tartary buckwheat are mainly selected individually from elite landraces, and the deficiency of genomic information and quantitative trait loci has until recently been the major limitation for modern breeding.

The first report of *Fagopyrum* genomics is a draft assembly of common buckwheat genome generated by next-generation sequencing (NGS), which was successfully used to identify novel candidate genes controlling heteromorphic self-incompatibility of common buckwheat [10]. Subsequently, a high-quality, chromosome-scale Tartary buckwheat genome sequence of 489.3 Mb was released, and 33,336 high-confidence genes were annotated based on expression evidence [11]. In this study, a worldwide collection of Tartary buckwheat germplasm resources, along with the availability of Tartary buckwheat genome sequence and enhanced capacity of sequencing, makes it possible to construct a comprehensive database of the genome variation. Here, we report the population structure and multiple domestication events of Tartary buckwheat by resequencing the genomes of 510 global accessions of wild and cultivated genotypes. The genome-wide association study (GWAS) was performed to identify key loci and genes associated with several important agronomic traits.

## Results

### Genome-wide variations and population structure

**Fig. 1:**
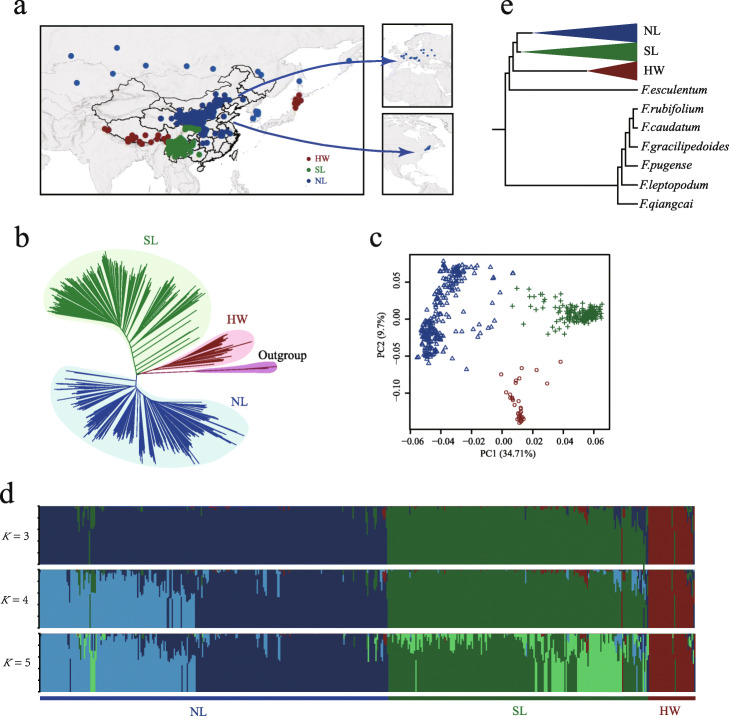
Geographic distribution and population structure of resequenced accessions from *Fagopyrum* species. **a** Geographic distributions of Tartary buckwheat accessions. Each accession is represented by a dot on the map. The spread routes are shown with blue lines, which represent from northern China to other countries. HW, Himalayan Wild accession; SL, Southwest Landraces; NL, North Landraces. **b** Neighbor-joining tree of 517 germplasms, including 510 Tartary buckwheat accessions and 7 other *Fagopyrum* species. Branch colors indicate different groups: group HW (red), group SL (green), group NL (blue), and outgroup (purple), matching the colors shown in **a**. **c** Principal component analysis of Tartary buckwheat accessions, showing the first two components. Colors correspond to the phylogenetic tree grouping. **d** The population structure analysis with different numbers of clusters (*K* = 3, 4, and 5) matches the phylogenetic tree. The *x* axis lists the different accessions that are consistent with those in the phylogenetic tree. **e** Phylogenetic tree of the outgroup species

We resequenced 510 worldwide Tartary buckwheat germplasm samples collected from 14 countries representing various geographical regions, including 483 landraces and 27 wild accessions, and 7 other *Fagopyrum* genus species (*F. esculentum*, *F. leptopodum*, *F. qiangcai*, *F. pugense*, *F. rubifolium*, *F. gracilipedoides*, *F. caudatum*) collected from Sichuan, China (Fig. 1a; Additional file 1: Figure S1; Additional file 2: Table S1). A total of 3.98 Tb of raw data were generated, with an average sequencing depth of 12.65× and 91.72% genome coverage. We identified a final set of 1,095,748 single-nucleotide polymorphisms (SNPs) (Additional file 2: Table S2) and 116,516 indels (1–50 bp in length) (Additional file 1: Figure S2; Additional file 2: Table S3). Site frequency spectrum (SFS) including folded and unfolded was applied to confirm the reasonableness of SNPs called from the whole-genome (Additional file 1: Figure S3). Most of the SNPs were located in intergenic regions and 2.3% were present in coding sequences, consisting of 8847 synonymous SNPs and 14,944 non-synonymous SNPs (Additional file 2: Table S2). This 1.69 ratio of non-synonymous to synonymous SNPs is higher than in pigeonpea (1.18) [12], chickpea (1.20) [13], castor (1.39) [14], and soybean (1.61) [15]. The Ts/Tv (transition/transversion) ratio of 2.175 (Additional file 2: Table S2) for Tartary buckwheat is higher than that for black gram (1.58) [16], tomato (1.75) [17], and maize (1.02) [18]. Variants exhibit potential large effects composed of 814 damaging SNPs causing the gain/loss of stop codons and 764 indels resulting in a frameshift. SNPs were validated using the PCR-based sequencing strategy, and the accuracy was estimated to be 95.1%, suggesting high reliability for SNP identification (Additional file 2: Table S4).

The phylogenetic analysis divided 510 Tartary buckwheat accessions into three major monophyletic clades (Fig. 1b; Additional file 1: Figure S4): group HW (Himalayan wild accessions) including 27 wild accessions and 9 landraces, group SL (Southwestern landraces) including 203 landraces, and group NL (Northern landraces) including 271 landraces. These results were further supported by principal component analysis (Fig. 1c) and model-based clustering analysis (Fig. 1d), which was as well consistent with DAPC analysis (Additional file 1: Figure S5). As expected, the phylogenetic tree suggested that Tartary buckwheat has a closer relationship to *F. esculentum*, the other cultivated buckwheat species, compared with other *Fagopyrum* genus species from the outgroup (Fig. 1e), which is consistent with the previous study [19].

The three groups displayed clearly different geographic distributions: wild accessions in HW mostly came from the Himalayan region; SL mainly included landraces from southwest China including Sichuan, Yunnan, and Guizhou provinces; and landraces from north and central China, Korea, Central Asia and Asian Russia, Europe, and North America were clustered in NL (Additional file 1: Figure S4; Additional file 2: Table S1). The genetic differentiation value (*F*_ST_) for Chinese and abroad individuals in NL (0.045) was much lower than the value for HW and abroad accessions of NL (0.16) (Additional file 2: Table S5). The phylogenetic map and *F*_ST_ values suggest a possible migration route of Tartary buckwheat from the northern China rather than the origin place to the other countries. Interestingly, only 3 accessions from Japan were found in cultivated groups (2 in NL and 1 in SL), and the rest (*n* = 7) were all grouped together with wild accessions, suggesting a direct import from Himalayan region to Japan besides the introduction from Russia, which was also recorded by Suzuki et al. [20, 21].

Cultivated groups SL and NL displayed significant advantages compared with the wild group HW in terms of plant development, yield, and quality traits, resulting from the artificial selection of favorable phenotypic traits (Additional file 1: Figure S6 and S7; Additional file 2: Table S6). Pairwise genome-wide *F*_ST_ values for HW with SL (0.173) and HW with NL (0.193) (Additional file 2: Table S5) indicated the notable genetic divergences between the wild group and each cultivated group, which was shaped by domestication.

### Independent domestication and divergence between SL and NL

**Fig. 2:**
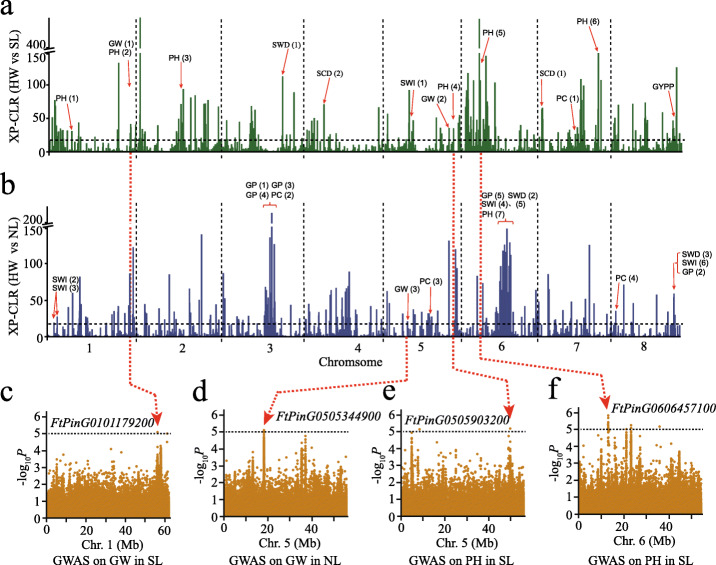
Genome-wide analysis of the independent selection in domestication traits between SL and NL. **a**, **b** Selective signals in domestication of SL (**a**, green) and in NL (**b**, blue) on the 8 chromosomes. Red arrows indicate highly selective genomic regions overlapped with GWAS signals. PH, plant height; GW, 1000-grain weight; GP, whole growth period; SCD, seed circular degree; SWI, seed wing; PC, pericarp color; GYPP, grain yield per plant. **c**, **d** Local Manhattan plots of GWAS signals overlapping with selection sweeps for GW in SL (**c**) and NL (**d**) populations. **e**, **f** Local Manhattan plots of GWAS signals overlapping with selection sweeps for PH on chromosome 5 (**e**) and 6 (**f**) in SL

To identify potential selective signals associated with Tartary buckwheat domestication, the cross-population composite likelihood ratio test (XP-CLR) was performed in the comparisons of HW versus SL and HW versus NL. Above the dashed horizontal thresholds of top 5%, we identified 150 sweeps between HW and SL containing 3415 putative genes (Fig. 2a; Additional file 2: Table S7), and 156 sweeps between HW and NL containing 3006 putative genes (Fig. 2b; Additional file 2: Table S8), which covered 8.0% (39 Mb) and 8.5% (41 Mb) of the assembled genome, respectively. It was notable that only 19 sweeps (4.1 Mb) and 420 genes overlapped for regions with selective signatures between SL and NL (Additional file 1: Figure S8), suggesting a likely independent domestication process driven by human intervention in the two genetically and geographically distinct groups. In addition to XP-CLR, de-correlated composite of multiple signals (DCMS) approach was also applied to detect selective sweeps. Above the dashed horizontal thresholds of top 10%, 146 sweeps (45 Mb) between HW and SL (Additional file 1: Figure S9a; Additional file 2: Table S9) and 112 sweeps (45 Mb) between HW and NL (Additional file 1: Figure S9b; Additional file 2: Table S10) were generated. Here, we identified 26.6 Mb (59%) and 30.6 Mb (68%) overlaps with sweeps identified by XP-CLR in SL and NL, respectively, indicating the reliability of two approaches.

To elucidate the genomic regions that underlie the remarkable phenotypical differences between SL and NL, we calculated the population differences and found 34 sweeps (top 5% *F*_ST_, Additional file 1: Figure S10a; Additional file 2: Table S11). The results from *F*_ST_ and the comparison of genetic diversity (π_wild_/π_landrace_) were combined to look for the unique selective sweep regions in SL and NL (Additional file 2: Table S12 and S13). We identified 4 unique selective sweeps in SL (Additional file 1: Figure S10b; Additional file 2: Table S14) and 8 in NL (Additional file 1: Figure S10c; Additional file 2: Table S14). The further exploitation and functional investigation of genes conferring genetic differentiation between SL and NL would contribute to the interpretation of Tartary buckwheat domestication process.

GWAS based on the Efficient Mixed-Model Association eXpedited (EMMAx) was performed for 10 traits to further determine the selected loci related to important agronomic traits during domestication (Additional file 1: Figure S9S11-S15S17). We found 31 GWAS signals that overlapped with selective sweeps identified by XP-CLR for plant height (PH), 1000-grain weight (GW), whole growth period (GP), seed circular degree (SCD), seed wing (SWI), seed width (SWD), pericarp color (PC), and grain yield per plant (GYPP) (Fig. 2a, b; Additional file 2: Table S15). GWAS analysis generated 34 signals that overlapped with selective sweeps identified by DCMS for GW, GP, seed length (SDL), SWD, SCD, SWI, PC, and PH (Additional file 2: Table S16). Comparing with XP-CLR, we found 22 overlapped GWAS signals (9 in SL and 13 in NL) for GW, GP, SWD, SCD, SWI, PC, and PH (Additional file 2: Table S16). The alignment with GWAS analysis and *F*_ST_ (Additional file 1: Figure S18-S21) generated 8 signals that are associated with GW, GP, SWD, SWI, and PC and overlapped with the divergent regions (Additional file 1: Figure S10a; Additional file 2: Table S17).

In two distinct selective sweeps of SL and NL associated with 1000-grain weight, we found a gene *FtPinG0101179200* on chromosome 1 (Fig. 2c), encoding the 13S globulin seed storage protein [22], and a gene *FtPinG0505344900* on chromosome 5 (Fig. 2d), encoding an auxin-induced protein. For plant height, 6 significant selective sweeps were found in SL and only 1 in NL. By further analysis of candidate genes related to plant growth in these association regions, we identified a protein kinase gene (*FtPinG0505903200*) in a selective sweep of SL on chromosome 5 (Fig. 2e), and its Arabidopsis homolog LRR kinase *AtVRLK1* was involved in switching between cell elongation and secondary cell wall thickening [23]. A translation factor gene related to plant height, *FtPinG0606457100*, was found on chromosome 6 in the selective sweep of SL as well (Fig. 2f), whose orthologous gene in Arabidopsis plays a crucial role in plant growth [24]. These genes were all confirmed via the factored spectrally transformed linear mixed models (FaST-LMM) (Additional file 1: Figure S22). These results supported that Tartary buckwheat underwent two independent domestication events, which was shaped by diverse genetic pathways in SL and NL, respectively.

### Genome-wide association with flavonoid metabolism

**Fig. 3:**
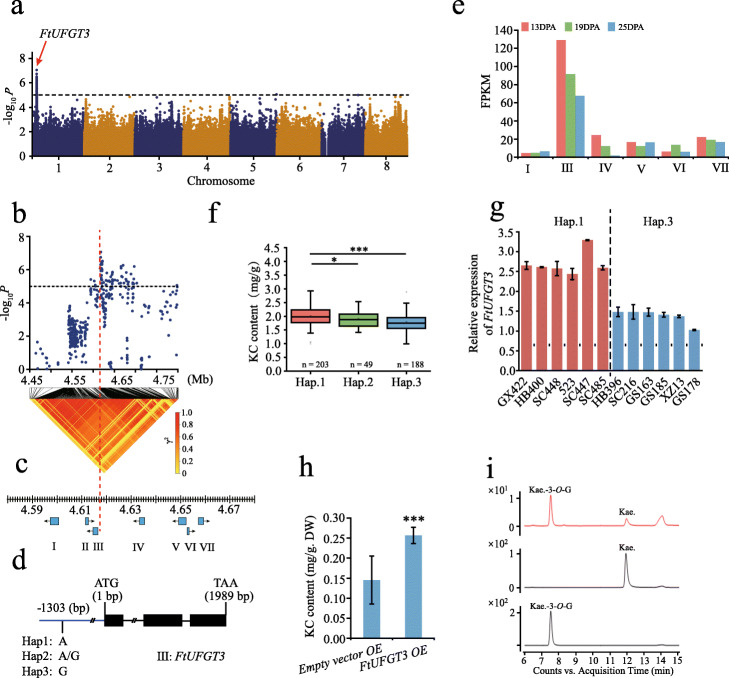
Identification of the *FtUFGT3* gene associated with kaempferol-3-*O*-rutinoside content. **a** Manhattan plot for GWAS on kaempferol-3-*O*-rutinoside content (KC) in the full population. The dashed line indicates the threshold −log*P* = 5. The red arrow indicates the SNP in *FtUFGT3*. **b** Local Manhattan plot (top) and linkage disequilibrium heat map (bottom) for the gene associated with KC. **c** Schematic representation of genes in the association region. I, *FtPinG0100123000*; II, *FtPinG0100123100*; III, *FtUFGT3*; IV, *FtPinG0100123600*; V, *FtPinG0100123800*; VI, *FtPinG0100123900*; VII, *FtPinG0100124100*. **d** Schematic representation of the *FtUFGT3* genomic sequence. Exons and introns are represented by boxes and lines, respectively. The blue line represents the promoter and 3′ UTR. The causal SNP locates in the promoter − 1303 bp that is marked by a black line. Hap1, haplotype 1; Hap2, haplotype 2; Hap3, haplotype 3. **e** Expression of genes from **c** during seed development excluding II. DPA, days postanthesis. **f** Box plots show KC in three haplotypes (Hap.). *P < 0.05, ***P < 0.001, Student’s *t* test. **g** Relative expression levels of *FtUFGT3* in different buckwheat accessions of Hap.1 and Hap.3. Data are mean ± SD. **h** KC in Tartary buckwheat transgenic hairy roots overexpressing *FtUFGT3*, mixed by five lines. Data are mean ± SD. ***P < 0.001, Student’s *t* test. **i** Enzyme assay of FtUFGT3 in vitro. Up for reaction product of MBP-FtUFGT3 protein and kaempferol standard; middle for kaempferol standard; below for kaempferol-3-*O*-glucoside standard. Kae., kaempferol

The richness of flavonol compounds appears as one of the most prominent healthy and pharmaceutic properties of Tartary buckwheat. Here, we employed GWAS analysis by both FaST-LMM and EMMAx models for 480 accessions to identify potential genes that are prominently correlated with the contents of three flavonols (Additional file 1: Figure S23-S25), including quercetin (QC), rutin (RC), and kaempferol-3-*O*-rutinoside (KC). One significant association with the kaempferol-3-*O*-rutinoside content was identified on chromosome 1 (Fig. 3a; Additional file 1: Figure S24; Additional file 2: Table S18), and 20 candidate genes were found in this association region (4.52–4.72 Mb) (Fig. 3b, c; Additional file 2: Table S19). The peak SNP (Ft1:4617722, A/G) generated three haplotypes, Hap.1 (AA), Hap.2 (AG), and Hap.3 (GG), and located at the promoter of *FtUFGT3* (*FtPinG0100123400*) gene (Fig. 3d), encoding an UDP-glucosyltransferase that can catalyze anthocyanin to anthocyanin-3-*O*-glucoside [25]. The expression of *FtUFGT3* in seeds was remarkably higher than the other candidate genes and decreased during the seed maturation from 13 to 25 DPA (days postanthesis) (Fig. 3e). We further found that the Hap.1 correlated with higher kaempferol-3-*O*-rutinoside content and that the Hap.3 correlated with lower content (Fig. 3f). The subsequent test of the *FtUFGT3* gene expression showed obviously the positive correlation with kaempferol-3-*O*-rutinoside accumulation (Fig. 3g). Overexpression of *FtUFGT3* in Tartary buckwheat hairy roots achieved elevated content of kaempferol-3-*O*-rutinoside in vivo (Fig. 3h; Additional file 1: Figure S26), and in vitro enzyme assays showed that FtUFGT3 catalyzed kaempferol into kaempferol-3-*O*-glucoside (Fig. 3h; Additional file 1: Figure S27). These results demonstrated that FtUFGT3 was involved in flavonoid metabolism.

For the quercetin content (QC), we found a candidate gene, *FtPinG0100487500* (Additional file 1: Figure S25; Additional file 2: Table S20), encoding a glutathione transferase, whose orthologs in Arabidopsis (*TT19*) [26], tea (*CsGSTa, b, c*) [27], and peach (*PpGST1*) [28] have been reported to be essential for the storage of anthocyanins, flavonols, and proanthocyanidins. Pericarp color (PC) shaped by the accumulation and oxidation of flavonoids is also one of the key quality traits during artificial selection for different use and preference. We identified a significant GWAS signal on chromosome 2 using the black and non-black accessions (Additional file 1: Figure S28a), and the peak SNP (Ft2:44531080 C/G) yielded three haplotypes, Hap.1 (GG), Hap.2 (CG), and Hap.3 (CC). More than 90% of Hap.1 gathered in the black seeds and 99% of Hap.3 exhibited non-black type (Additional file 1: Figure S28b), indicating the significant correlation between Hap.1 and black pericarp color. Among the 9 genes located in this association region (44.43–44.63 Mb), the *FtPinG0202040000* gene (Additional file 1: Figure S28c; Additional file 2: Table S20) is homologous to Arabidopsis *TT2* that encodes a MYB transcription factor well-known as the key positive regulator of proanthocyanidin accumulation in developing seed [29]. In summary, all these genetic loci and candidate genes can be potentially used to improve the quality of buckwheat.

### Genome-wide association with agronomic traits

**Fig. 4:**
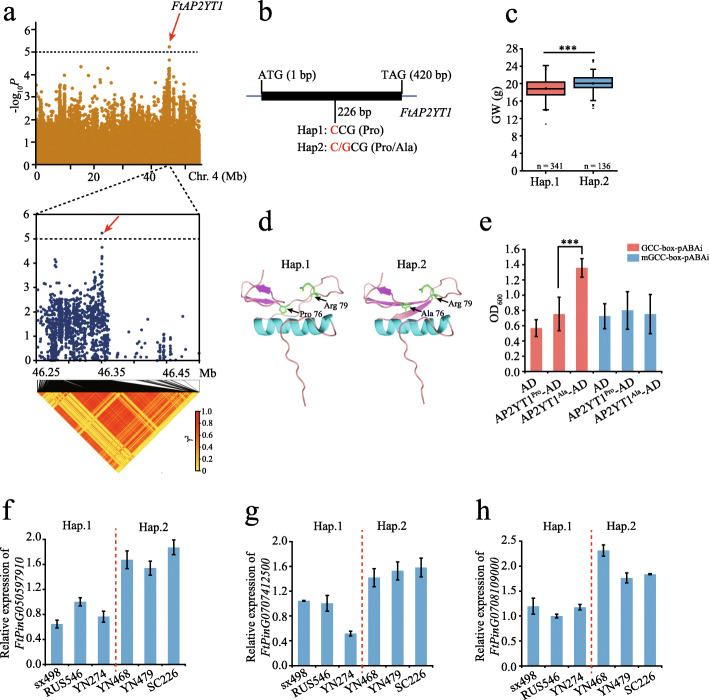
Identification of the candidate gene *FtAP2YT1* associated with 1000-grain weight. **a** Manhattan plot for GWAS on 1000-grain weight (GW). The dashed line indicates the threshold −log*P* = 5. The red arrow indicates the SNP in *FtAP2YT1*. **b** Structure of *FtAP2YT1* genomic sequence containing only one exon that is represented by a box. The non-synonymous SNP at 226 bp is marked by a black line. Hap1, haplotype 1; Hap2, haplotype 2. **c** Box plots show GW in two haplotypes (Hap.). ***P < 0.001, Student’s *t* test. **d** Protein structure model of FtAP2YT1. Compared with site Pro76, Ala76 enlarged the β-sheet length, which functions in binding to the DNA. **e** Y1H analysis of the activity of FtAP2YT1^Pro^ and FtAP2YT1^Ala^ binding to GCC-box and mGCC-box. Data are mean ± SD. ***P < 0.001, Student’s *t* test. **f**–**h** Expression of genes containing GCC-box that are potential targets of FtAP2YT1 in different accessions carrying Hap.1 and Hap.2, respectively

The agronomic characters, such as yield and growth period, always come to the first in crop breeding, of which the regulatory mechanism is complicated. Using GWAS (FaST-LMM and EMMAx models) for 1000-grain weight that quantifies the yield directly (Additional file 1: Figure S29), we identified 2 non-synonymous SNPs that were located at *FtPinG0404616900* (C/G, Ft4:46350596) and *FtPinG0280000714* (T/G, Ft2:37407976), which encode an AP2 transcription factor and an unknown protein, respectively (Additional file 2: Table S21 and S22). Therefore, *FtPinG0404616900* (*FtAP2YT1*) was chosen for further functional investigation as the candidate gene associated with the 1000-grain weight trait (Fig. 4a). We identified 2 haplotypes, Hap.1 (CC) correlating with lower grain weight and Hap.2 (CG) correlating with higher 1000-grain weight (Fig. 4b, c). This non-synonymous SNP (C/G) led to an amino acid change from Pro to Ala at the position of the second β-sheet, where it is close to its DNA binding domain [30] (Fig. 4d). Yeast one-hybrid assay showed a prominently increased binding activity of FtAP2YT1^Ala^ to the GCC *cis*-element compared to FtAP2YT1^Pro^ (Fig. 4e; Additional file 1: Figure S30). We further selected 7 potential target genes of FtAP2YT1 with the GCC *cis*-element that correlated with grain weight (Additional file 2: Table S23). The expression of three genes, *FtPinG0505979100*, *FtPinG0707412500*, and *FtPinG0708109000*, was significantly higher in accessions of higher grain weight carrying Hap.2 than that of lower grain weight carrying Hap.1 (Fig. 4f–h). These results indicate that FtAP2YT1 may play an important role in regulating the expression of genes involved in 1000-grain weight.

Seed size is also an important factor that determines crop yield. Our GWAS analysis identified 2 candidate genes associated with seed width, including the above gene (*FtPinG0404616900*) related to 1000-grain weight (Additional file 1: Figure S31; Additional file 2: Table S20). *FtPinG0100980400* encodes a tryptophan aminotransferase-related protein that has been reported to be critical for grain yield via auxin biosynthesis in rice [31] and wheat [32]. Candidate genes in the important agronomic traits associated regions can be potentially used for the yield improvement of Tartary buckwheat cultivars.

## Discussion

The rich genetic diversity of *Fagopyrum* resources and the characterization of cultivated buckwheat ancestors in Himalayan region suggest the possible origin of cultivated buckwheat. The diffusion of buckwheat was then proposed that it was brought from China and southern Himalaya to the world after the birth of cultivated buckwheat [3, 33]. In our research, the closely phylogenetic relationship between accessions from northern China and from outside China (Additional file 1: Figure S4), along with the *F*_ST_ value (Additional file 2: Table S5), provides evidences for the hypothesis that Tartary buckwheat was introduced from the northern China to the other countries after wild Tartary buckwheat was domesticated in China. Given the lack of archeological and palynological evidences and small number of wild accessions, this result has limited capacity to unravel the historical migration of Tartary buckwheat.

After the cultivation and artificial intervention for thousands of years, the 1000-grain weight increased and the whole growth period decreased significantly in cultivated Tartary buckwheat accessions comparing with wild accessions. However, unlike most gramineous and legume crops, this pseudocereal still possesses wild characterizations, including seed shattering, thick shell, tall stalk, plant lodging, and low yield. Besides the phenotypes, well-characterized domestication genes, such as *sh4* for seed shattering of rice [34], *tga1* for naked grains of maize [35], PROG1 for tiller angle of rice [36], and *G* for seed dormancy in soybean, rice, and tomato [37], were not found in the genetically selective sweeps obtained by multiple methods, supporting that the domestication process is still at the very early stage.

SL and NL landraces showed a clear geographic and genetic division, besides the phenotypic differences between the two groups, including seed size, plant height, whole growth period, and grain weight. The divergence between SL and NL landraces can be explained by the artificial selection for locally adapted and preferred traits. Those unique selective sweep regions and their underlying genes in SL and NL generated from the independent domestication process (Fig. 2; Additional file 1: Figure S18; Additional file 2: Table S15 and S16) provide potential valuable resources for molecular breeding of Tartary buckwheat. For instance, a protein kinase gene *FtPinG0505903200* associated with plant height was identified in a selective sweep of SL (Fig. 2). This gene belongs to the leucine-rich repeat receptor-like protein kinase (LRR-RLK) family that is the largest group of receptor-like kinases in plants and plays crucial roles in development and stress responses [38]. Therefore, Tartary buckwheat LRR-RLKs were extracted and their conserved kinase domains were aligned with Arabidopsis LRR-RLKs. In total, the 199 buckwheat LRR-RLKs were divided into 19 different subfamilies and the *FtPinG0505903200* was clustered in the VIII-1 group (Additional file 1: Figure S32), which can provide insights into possible gene functions and mechanisms of functional divergence.

## Conclusion

In the present study, from the abundant genome variations of Tartary buckwheat accessions including wild samples and landraces, we achieved genomic regions that have experienced selective sweeps corresponding to separate domestication events of SL and NL. Unique selective sweeps and candidate genes in the two geographically different groups might be useful for modern molecular breeding. In addition, using GWAS, we identified several genomic loci that contribute to the formation of important quality and yield traits of Tartary buckwheat. Functional analysis of a key enzyme gene *FtUFGT3* associated with flavonoid metabolism and a transcription factor *FtAP2YT1* associated with grain weight displayed their potential use on the improvement of elite Tartary buckwheat cultivars. All these loci generated by GWAS analysis conferred promising candidates for not only buckwheat but also other crop improvement.

In summary, this research provides extensive genomic resources to gain insights into the buckwheat origin, spread, and domestication and will promote future breeding in character improvement, especially in yield and quality.

## Methods

### Plant materials and phenotyping

For genome resequencing, we used 510 Tartary buckwheat accessions composed of 32 wild accessions, 478 landraces, and 7 other *Fagopyrum* genus species (*F. esculentum*, *F. leptopodum*, *F. qiangcai*, *F. pugense*, *F. rubifolium*, *F. gracilipedoides*, *F. caudatum*) as the outgroup, which were obtained from holdings of the National Crop Genebank of China (NCGC) and collections of worldwide buckwheat research groups. These accessions are mainly from 20 provinces in China as well as from South Korea, Japan, Russia, Poland, USA, Nepal, Bhutan, India, Slovenia, France, and Belgium, covering most areas where Tartary buckwheat is currently cultivated (Additional file 2: Table S1).

For phenotyping, a total of 480 Tartary buckwheat accessions were grown in Liangshan (Sichuan province, 27° 59′ N, 102° 50′ E) and Zhaotong (Yunnan province, 28° 36′ N, 103° 49′ E) in 2017 and 2018. All seeds were sown by hand in three replications on 12 April, and seeds were harvested in the middle of July. Three individual plants from each accession in each replication were used for measurements of plant height (PH). The whole growth period (GP) was defined as the growing days from sowing to maturity. Mature seeds were harvested from the selected six plants for measurements of 1000-grain weight (GW), grain yield per plant (GYPP), seed length (SLE), seed width, (SWD) seed circular degree (SCD), seed wing (SWI), pericarp color (PC), and seed diameter (SD).

### Measurement of flavonoid content

Seeds were smashed and filtered by the 40-mesh sieve after they were pre-dried at 105 °C for 30 min and kept at 65 °C to a constant weight. Samples (0.2 g) were ultrasonically extracted in 20 ml 80% methanol at 50 °C and 40k Hz for 25 min. The solution was then filtered through the 0.22-μm organic microporous filter and analyzed by high-performance liquid chromatograph (HPLC, Agilent G6500 Series HPLC-QTOF). A RP18 column (2.1 mm × 75 mm × 2.7 μm) was operated at 40 °C. The mobile phase consisted of a mixture of (A) water/formic acid (99.9/0.1, v/v) and (B) methanol/formic acid (99.9/0.1, v/v). The gradient program was set as follows: 0–13 min, 20% (B); 13–13.5 min, gradually rose to 50% (B); 13.5–17 min, gradually decrease to 20% (B); 17–18 min, keep 20% (B); and 18.1 min, stop. The contents of rutin (RC), quercetin (QC), and kaempferol-3-*O*-rutinoside (KC) were calculated by comparing the HPLC peak area with authenticated standards (Sigma-Aldrich, USA). Three replicates were performed for every sample.

### DNA extraction and sequencing

Genomic DNA was extracted from young leaves using the cetyltrimethylammonium bromide (CTAB) method. At least 1 μg genomic DNA for each accession was used to construct a sequencing library according to vendor-provided instructions (Illumina). Paired-end sequencing libraries with an insert size of approximately 350 bp were sequenced on an Illumina NovaSeq 6000 platform in Berry Genomics. Trimmomatic v0.33 was used to trim the Illumina fastq and remove adapters based on the manufacturer’s adapter sequences. Raw data of fastq format were then processed through in-house perl scripts. In this step, clean data were obtained by removing reads containing adapter, reads containing poly-N, and low-quality reads from raw data.

### Read alignment and variant calling

All the sequenced reads for each accession were mapped to the assembly genome [11] (http://www.mbkbase.org/Pinku1/) using the Burrows-Wheeler Aligner program [39] (BWA 0.7.5a) with default parameters. We sorted the alignments according to mapping coordinates in samtools [40, 41] (0.1.19). After removing reads with low mapping quality (MQ < 30), both paired-end and single-end mapped reads were used for SNP detection across the entire sample set of buckwheat accessions using the GATK toolkit [42] (version 3.4-46-gbc02625). Reads having a mean of approximately 12× depth for each individual and > 70% mapping rate of the buckwheat genome were retained for SNP calling. SNPs and small indels (1–50 bp) were called using the GATK UnifiedGenotyper module for diploids with -stand_call_conf 50-stand_emit_conf 10-dcov 1000 to call variants. We filtered variants both per population and per individual using GATK according to stringent filtering criteria. For SNPs of population filter: (a) QUAL > 30.0; (b) QD > 5.0; (c) FS < 60.0; (d) MQ0 ≥ 4 && ((MQ0/(1.0*DP)) > 0.1); (e) DP > 5. We are working on a non-model organism and there is no SNP data available, so following GATK best practice tutorial, we chose Hard Filtering instead of Variant Recalibration (VQSR) method to filter our variants callset (https://gatk.broadinstitute.org/hc/en-us/articles/360036434492-VariantFiltration). According to the GATK toolkit, if there were more than 3 SNPs clustered in a 10-bp window, all three SNPs were considered as false positives and removed according to the GATK toolkit. All SNPs and indels were assigned to specific genomic regions and genes using ANNOVAR [43] based on buckwheat genome annotations. To make sure that SNPs called from the whole-genome resequencing data are reasonable, site frequency spectrum (SFS) was applied [44] with the callset at population level based on MAF > 0.05 and missing rate < 0.1.

### SNP validation

A total of 510 SNPs were selected randomly and detected by PCR-based sequencing in 10 Tartary buckwheat accessions, using PCR-based sequencing in more than 3 replicates (Additional file 2: Table S4). We aligned all the PCR products against the reference genome with DNAMAN software.

### Phylogenetic and population structure analyses

We removed all SNPs with a minor allele frequency ≤ 0.05 and a missing rate > 10% in all accessions. A subset of 1,094,031 SNPs were used for phylogenetic and population structure analysis. Vcf files were converted to hapmap format with custom perl scripts and to PLINK format file by PLINK v1.90 (http://pngu.mgh.harvard.edu/purcell/plink/). Under the p-distances model with bootstrapping (100), a neighbor-joining tree of all samples was constructed with TreeBest 1.9.2 [45]. SNPRelate [46] (1.18.1) was used to carry out principal component analysis (PCA), first by generating the genetic relationship matrix from which the first 3 eigenvectors were extracted. fastStructure [47] (version 1.0) was used for inferring population structure from large SNP genotype data sets. *K* values were set from *K* = 3 to *K* = 5. Each *K* value, as a putative number of populations set from 1 to 10, was obtained with five independent runs with different starting seeds. The length of the burn-in period and number of MCMC replications after burn-in were set to 50,000 and 100,000, respectively. The optimum number of subgroups (*K*) was determined based on the log probability of the data (lnP(*K*)) and an ad hoc statistic Δ*K* method. To further confirm the result of the structure, the discriminant analysis of principal components (DAPC) was used to cluster genotypes independently of a priori haplotype designation using the R package adegenet v. 1.4.2 [48].

### Identification of selective sweeps

To detect selective sweeps, the cross-population composite likelihood ratio test XP-CLR v1.0 [49] was performed. We compared HR with SL and HR with NL group in 200-kb sliding windows with a step size of 100 kb. The highest XP-CLR values, accounting for 5% of the genome, were considered as selected regions. Adjacent windows with high XP-CLR were grouped into a single region to represent the effect of a single selective sweep. In addition to XP-CLR, de-correlated composite of multiple signals (DCMS) approach [50] was performed and the comparison of genetic diversity (π_wild_/π_landrace_) was calculated to identify selective sweeps using the same 200–100-kb sliding window.

### Identification of genomic differentiation

The level of genetic differentiation (*F*_ST_) between populations was calculated in 200-kb intervals using PopGenome [51]. To detect differentiated regions, the average *F*_ST_ of all sliding windows across group II and NM were compared.

### GWAS and identification of the candidate genes

Only SNPs with MAF ≥ 0.05 and missing rate ≤ 0.1 were used for GWAS, which resulted in 844,290, 885,276, and 1,094,031 SNPs for SL, NL, and the entire population (SL, NL, and HW), respectively. The association analysis was done with the Efficient Mixed-Model Association eXpedited program (EMMAx) [52] and the factored spectrally transformed linear mixed models (FaST-LMM) [53]. The effective number of independent SNPs was estimated as 988,845, and thus, the significance threshold was estimated approximately *P* = 10^−6^.

According to the associated loci determined by GWAS, SNP types and locations were identified using the reference genome [11]. The total genes in each candidate region were analyzed and annotated by homologous comparison with Arabidopsis to narrow down the candidate genes.

### Enzyme assays

The *FtUGT3* CDS was inserted into the pMAL-C2X MBP [54] fusion expression vector and transformed into *E. coli* BL21. The MBP fusion proteins were extracted and immobilized onto amylose beads (New England Biolabs) with protein extraction buffer (20 mM Tris-HCl (pH 7.4), 0.2 M NaCl, and 1 mM EDTA). Protein was eluted using 20 mM Tris-HCl (pH 7.4), 0.2 M NaCl, 1 mM EDTA, and 10 mM maltose.

The reaction mixture (pH 8.0, 100 mM Tris-HCl, 14 mM β-mercaptoethanol, 9 mM UDP-glucose, and 100 μM kaempferol) was added to 5 μg purified protein and incubated at 37 °C for 30 min. The reaction was terminated by the freeze-dryer at − 40 °C. The dried reaction products were re-dissolved in 80% methanol, and 5 μl of the solution was analyzed by LC-MS (Agilent G6500 Series HPLC-QTOF) to determine the product using the standards of kaempferol and kaempferol-3-*O*-glucoside (Sigma-Aldrich, USA).

### Transgenic hairy roots

The *FtUGT3* CDS was inserted into the pCAMBIA 1307 vector and transformed into *Agrobacterium* A4 to generate transgenic hairy roots following previous methods [55–57]. Two weeks old sterile seedlings of Tartary buckwheat were cut and used as the explant for the infection with the *Agrobacterium* for 10 min. After the co-culture on MS solid medium in the dark for 48 h at 25 °C, explants were washed by MS liquid medium containing 300 mg/ml cefotaxime and sterile water, and then cultured on MS solid medium containing 300 mg/ml cefotaxime in the growth chamber for the hairy root induction. The induced single hairy root lines were removed from explants after 2 weeks and put on MS solid medium with 100 mg/ml cefotaxime for detoxification and growth. The positively transgenic lines were tested by PCR and moved to MS liquid medium with 100 mg/ml cefotaxime shaking for 2 weeks in the dark at 22 °C, 160 r/min. Hairy roots were then harvested and dried for the measurement of kaempferol-3-*O*-rutinoside as described above.

### Yeast one hybrid (Y1H)

The *cis*-elements GCC-box (AGTGCCAAAAGCCGCCACACCCCT) and mGCC-box (AGTGCCAAAATCCACTACACCCCT) were inserted into pABAi vector as reporters, respectively. The reporters were linearized using restriction enzyme BbsI and transformed into Y1H gold strain. *FtAP2YT1*^*Pro*^ and *FtAP2YT1*^*Ala*^ were inserted into pGADT7 vector containing a GAL4 transcriptional activation domain as effectors, respectively. The effectors were transformed into the Y1H gold strain containing the reporter gene, respectively. Transformants were plated on minimal synthetic defined (SD)-glucose medium lacking Leu (-L) and selected on SD-L medium with Aureobasidin A. Y1H assay was performed according to the manufacturer’s protocol (Matchmaker One-Hybrid System; Clontech; http://www.clontech.com/).

### Quantitative RT-PCR analyses

Total RNA of 7-day-old buckwheat seedlings were extracted by RNApre Pure Plant Plus Kit (DP441, Tiangen, Beijing, China). Reverse transcription was carried out using the HiScript III RT SuperMix for qPCR (R323-01, Vazyme, Nanjing, China) according to the manufacturer’s protocol. The qRT-PCR was performed as the protocol of ChamQ Universal SYBR qPCR Master Mix (Q711, Vazyme, Nanjing, China).

## Supplementary Information

**Additional file 1: Figure S1.** *Fagopyrum* species used in this research. **Figure S2.** Distribution of small indels. **Figure S3.** Site frequency spectrum for all accessions. **Figure S4.** Neighbor-joining tree analysis of 517 buckwheat accessions (HW in red, SL in green, NL in blue) using SNPs detected in whole-genome resequencing data. **Figure S5.** Population structure of Tartary buckwheat with DAPC. **Figure S6** and **Figure S7.** Quantification of agronomic and quality traits of 480 accessions in three groups. **Figure S8.** Venn diagram for the overlap of selective sweeps (a) and genes (b) correlated with independent domestication between SL and NL. **Figure S9.** Genome wide analysis of selection sweeps during independent domestication used by de-correlated composite of multiple signals (DCMS). **Figure S10.** Unique selective sweep regions analysis with FST and the comparison of nucleotide diversity. **Figure S11.** GWAS analysis of 1000-grain-weight in SL and NL, respectively. **Figure S12.** GWAS analysis of seed width in SL and NL, respectively. **Figure S13.** GWAS analysis of seed circular degree in SL and NL, respectively. **Figure S14.** GWAS analysis of whole growth period in SL and NL, respectively. **Figure S15.** GWAS analysis of plant height in SL and NL, respectively. **Figure S16** and **Figure S17.** GWAS analysis of agronomic traits in SL and NL, respectively. **Figure S18** to **S21.** GWAS analysis of differentiation traits. **Figure S22.** GWAS analysis of GW and PH in SL and NL used by FaST-LMM, respectively. **Figure S23.** GWAS analysis of rutin content. **Figure S24.** GWAS analysis of kaempferol-3-*O*-rutinoside content. **Figure S25.** GWAS analysis of quercetin content. **Figure S26.** Hair root transgenic system in buckwheat. **Figure S27.** In vitro enzyme assay of FtUFGT3. **Figure S28.** GWAS analysis of pericarp color. **Figure S29.** GWAS analysis of 1000-grain-weight. **Figure S30.** Y1H assay for the activity of FtAP2YT1^Pro^ and FtAP2YT1^Ala^ binding to GCC-box and mGCC-box. **Figure S31.** GWAS analysis of seed width. **Figure S32.** Maximum likelihood tree of LRR-RLK genes.

**Additional file 2: Table S1.** Summary of all accessions sequenced in this study. **Table S2.** Distribution of SNPs within various genomic regions in Tartary buckwheat. **Table S3.** Distribution of indels within various genomic regions. **Table S4**. The validation of random selected SNPs by PCR methods. **Table S5.** Levels of genetic differentiation in different chromosomes. **Table S6.** 13 agronomic and quality traits used in GWAS. **Table S7.** Putative regions and genes experiencing domestication sweep between HW and SL. **Table S8.** Putative regions and genes experiencing domestication sweep between HW and NL. **Table S9.** Putative regions and genes experiencing domestication sweep between HW and SL calculated by DCMS. **Table S10.** Putative regions and genes experiencing domestication sweep between HW and NL calculated by DCMS. **Table S11.** Putative regions and genes experiencing differentiation sweep between SL and NL. **Table S12.** Putative regions and genes experiencing domestication sweep between HW and SL. **Table S13.** Putative regions and genes experiencing domestication sweep between HW and NL. **Table S14.** Regions identified by *F*st approaches overlapped with the SL or NL unique selective sweep regions. **Table S15.** Overlaps between domesticated regions and GWAS signals for all traits. **Table S16.** Overlaps between domesticated regions and GWAS signals for all traits calculated by DCMS. **Table S17.** Overlaps between differentiated regions and GWAS signals for all traits. **Table S18.** SNP analysis associated with Ft1:4617722 for kaempferol-3-*O*-rutinoside content (-log*P* > 5). **Table S19.** Identification of candidate genes associated with Ft1:4617722 on Chr.1 for kaempferol-3-*O*-rutinoside content. **Table S20.** Information of the candidate genes from GWAS analysis. **Table S21.** Total SNPs for GWAS on 1000-gain-weight (-log*P* > 5). **Table S22.** Identification of candidate genes associated with Ft4:46350596 on Chr.4 for 1000-gain-weight. **Table S23.** The information of *FtPinG0404616900* potential target genes from GWAS on 1000-gain-weight. **Table S24.** Primers used in this study.

**Additional file 3.** Review history.

## Data Availability

All genomic sequence raw data sets for genetic diversity analysis and GWAS are available from NCBI under BioProject accession no. PRJNA600676 [58]. The scripts, codes, and analytic tools are deposited at the GitHub repository (https://github.com/rahello/tartary_population) [59] and Zenodo with the access code DOI: 10.5281/zenodo.3972746 [60].
