# Supplementary material for: Resequencing of global Tartary buckwheat accessions reveals multiple domestication events and key loci associated with agronomic traits
Source: Genome Biol. 2021 Jan 12;22:23. doi: 10.1186/s13059-020-02217-7 (PMC7802136; doi:10.1186/s13059-020-02217-7)
Supplement: Supplementary file 1 — Additional file 1: Figure S1. Fagopyrum species used in this research. Figure S2. Distribution of small indels. Figure S3. Site frequency spectrum for all accessions. Figure S4. Neighbor-joining tree analysis of 517 buckwheat accessions (HW in red, SL in green, NL in blue) using SNPs detected in whole-genome resequencing data. Figure S5. Population structure of Tartary buckwheat with DAPC. Figure S6 and Figure S7. Quantification of agronomic and quality traits of 480 accessions in three groups. Figure S8. Venn diagram for the overlap of selective sweeps (a) and genes (b) correlated with independent domestication between SL and NL. Figure S9. Genome wide analysis of selection sweeps during independent domestication used by de-correlated composite of multiple signals (DCMS). Figure S10. Unique selective sweep regions analysis with FST and the comparison of nucleotide diversity. Figure S11. GWAS analysis of 1000-grain-weight in SL and NL, respectively. Figure S12. GWAS analysis of seed width in SL and NL, respectively. Figure S13. GWAS analysis of seed circular degree in SL and NL, respectively. Figure S14. GWAS analysis of whole growth period in SL and NL, respectively. Figure S15. GWAS analysis of plant height in SL and NL, respectively. Figure S16 and Figure S17. GWAS analysis of agronomic traits in SL and NL, respectively. Figure S18 to S21. GWAS analysis of differentiation traits. Figure S22. GWAS analysis of GW and PH in SL and NL used by FaST-LMM, respectively. Figure S23. GWAS analysis of rutin content. Figure S24. GWAS analysis of kaempferol-3-O-rutinoside content. Figure S25. GWAS analysis of quercetin content. Figure S26. Hair root transgenic system in buckwheat. Figure S27. In vitro enzyme assay of FtUFGT3. Figure S28. GWAS analysis of pericarp color. Figure S29. GWAS analysis of 1000-grain-weight. Figure S30. Y1H assay for the activity of FtAP2YT1Pro and FtAP2YT1Ala binding to GCC-box and mGCC-box. Figure S31. GWAS analysis of seed width. Figure S32. Maxim [file 13059_2020_2217_MOESM1_ESM.pdf]

Figure S1

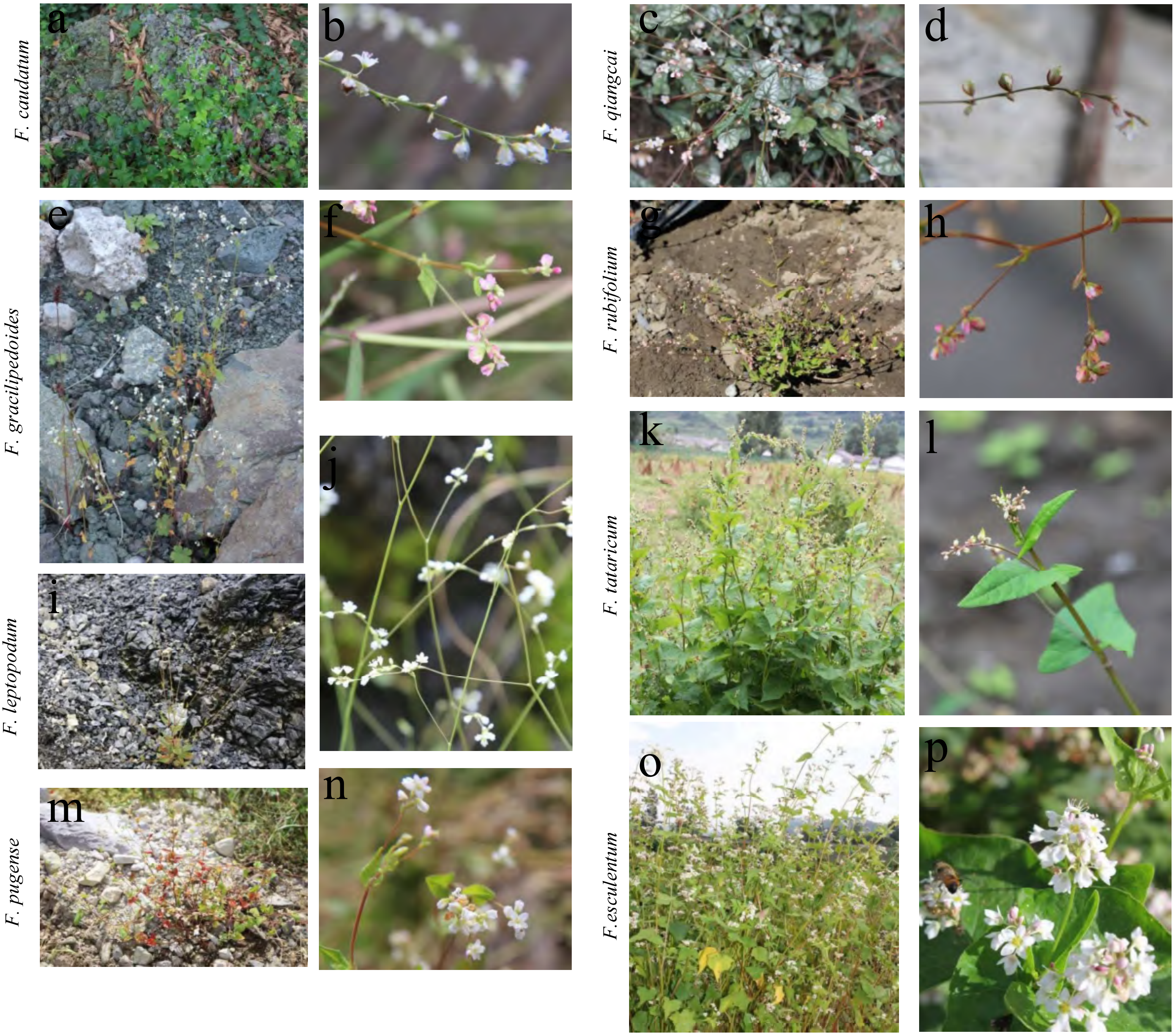
F. caudatum
a
b
F. qiangcai
c
d
F. gracilipedoides
e
f
F. rubifolium
g
h
F. leptopodum
i
j
F. tataricum
k
l
F. pugense
m
n
F.esculentum
o
p

Figure S1 *Fagopyrum* species used in this research. a,b, *F. caudatum*; c,d, *F. qiangcai*; e,f, *F. gracilipedoides*; g,h, *F. rubifolium*; i,j, *F. leptopodum*; k,l, *F. tataricum*; m,n, *F. pugense*; o,p, *F. esculentum*.

Figure S2

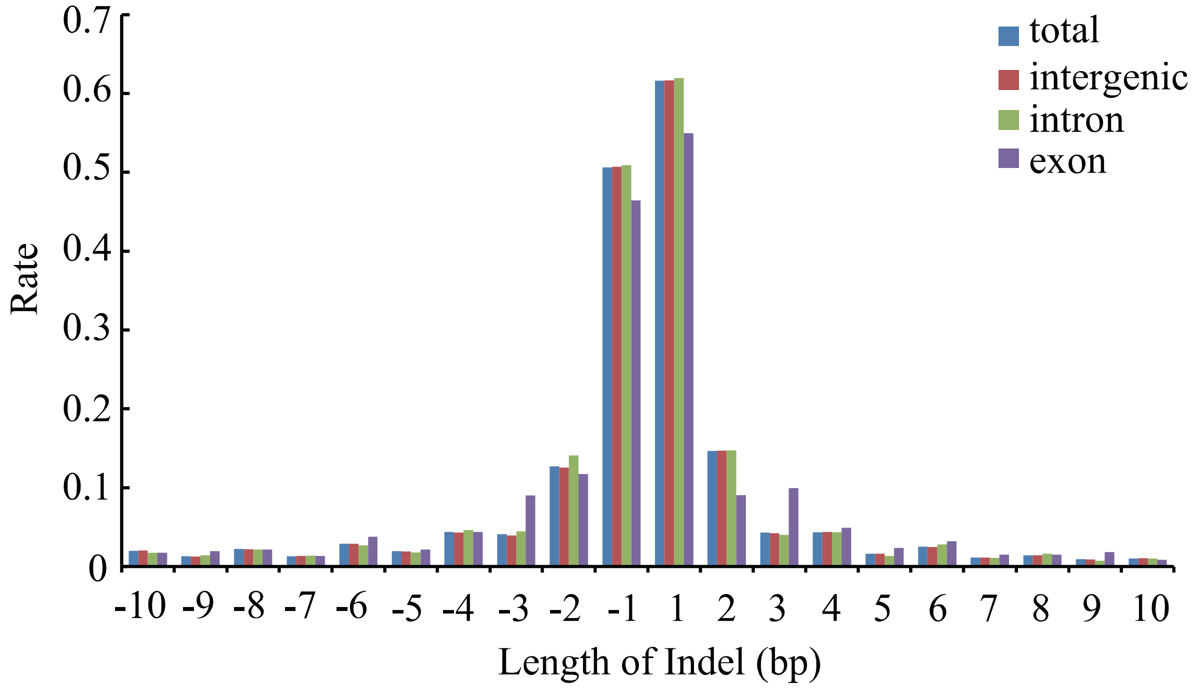
total
intergenic
intron
exon
Rate
0.7
0.6
0.5
0.4
0.3
0.2
0.1
0
-10
-9
-8
-7
-6
-5
-4
-3
-2
-1
1
2
3
4
5
6
7
8
9
10
Length of Indel (bp)

Figure S2 Distribution of small indels. Distribution of small indels (≤10bp) in different genomic regions. Indels in all regions were shown in blue, intergenic regions were in red, introns were in green and exons in purple.

Figure S3

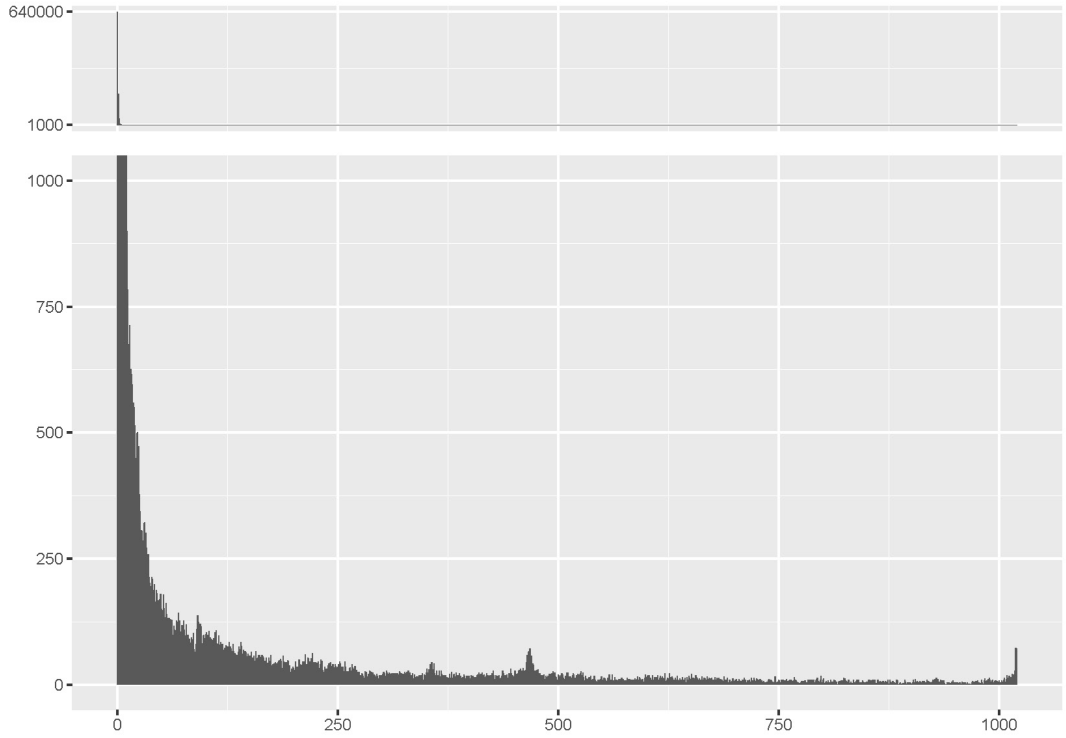

Figure S3 Site frequency spectrum for all accessions.

Figure S4

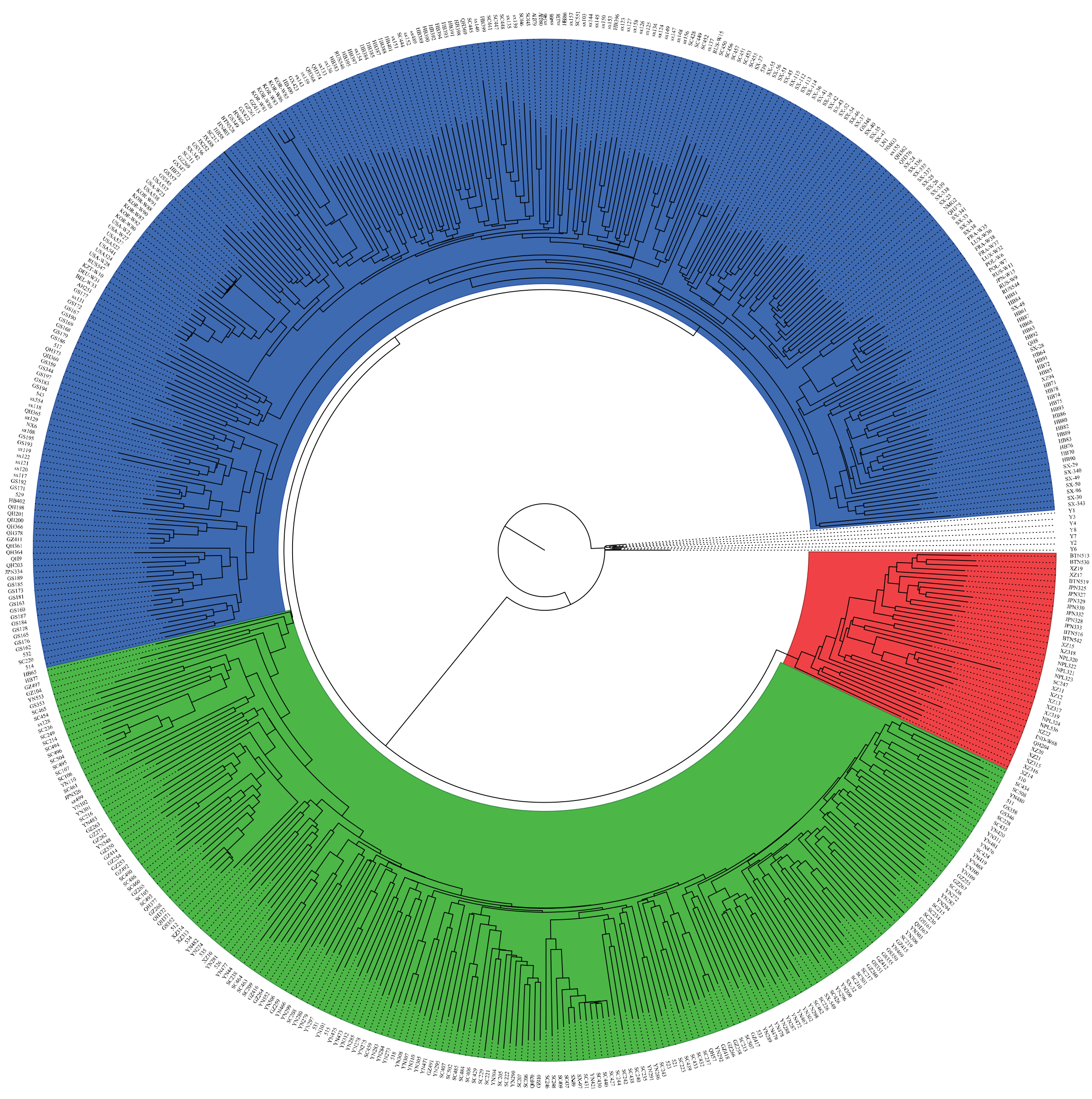

Figure S4 Neighbor-joining tree analysis of 517 buckwheat accessions (HW in red, SL in green, NL in blue) using SNPs detected in whole-genome resequencing data. Accession names are given in the out ring.

Figure S5

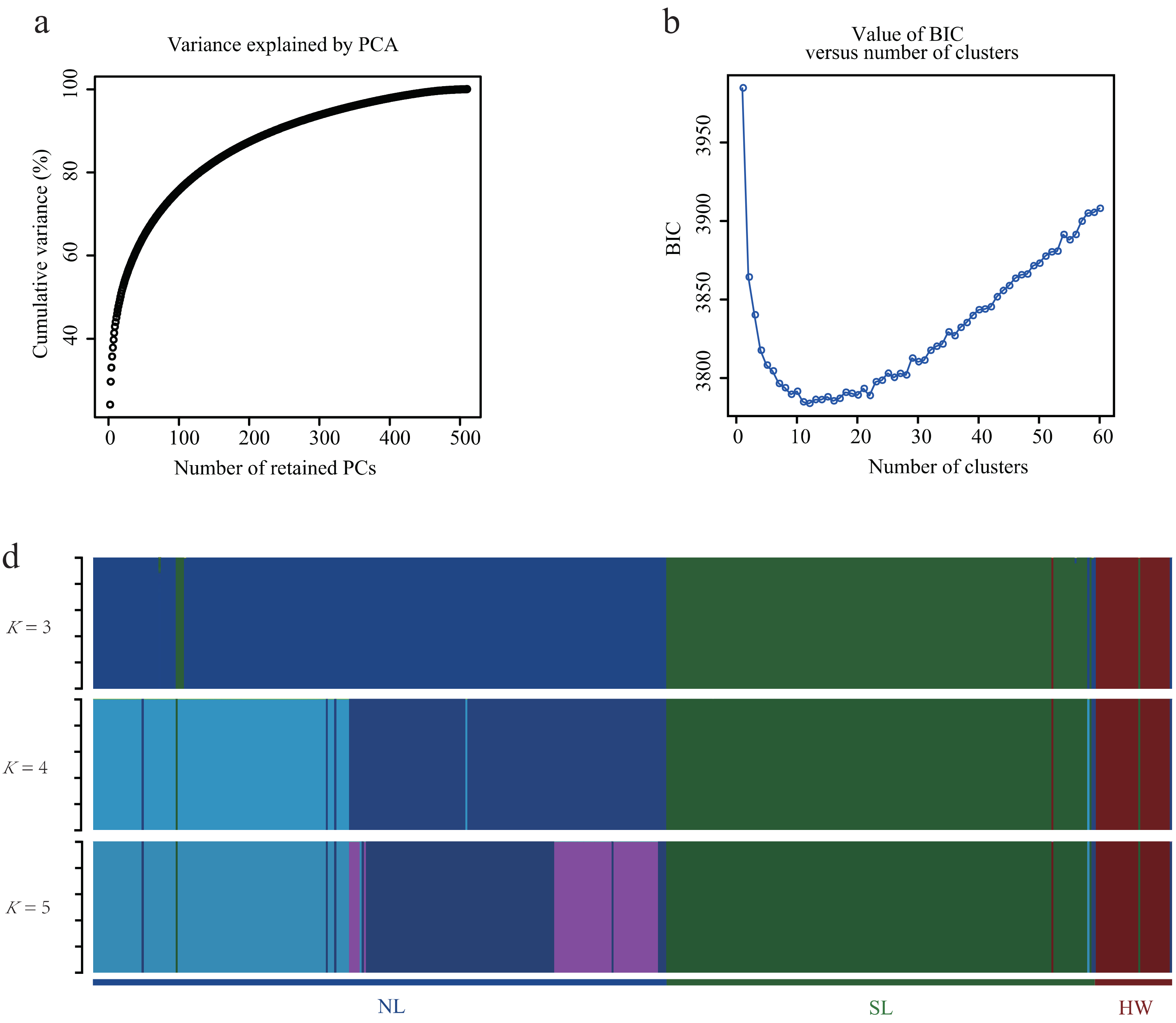

a
Variance explained by PCA
Cumulative variance (%)
Number of retained PCs
b
Value of BIC
versus number of clusters
BIC
Number of clusters
d
K = 3
K = 4
K = 5
NL
SL
HW

Figure S5 Population structure of Tartary buckwheat with DAPC. a, Cumulated variance explained by the eigenvalues of the PCA. b, Variation curve of BIC value. c, Model-based clustering analysis with different numbers of clusters (K=3, 4 and 5).

Figure S6

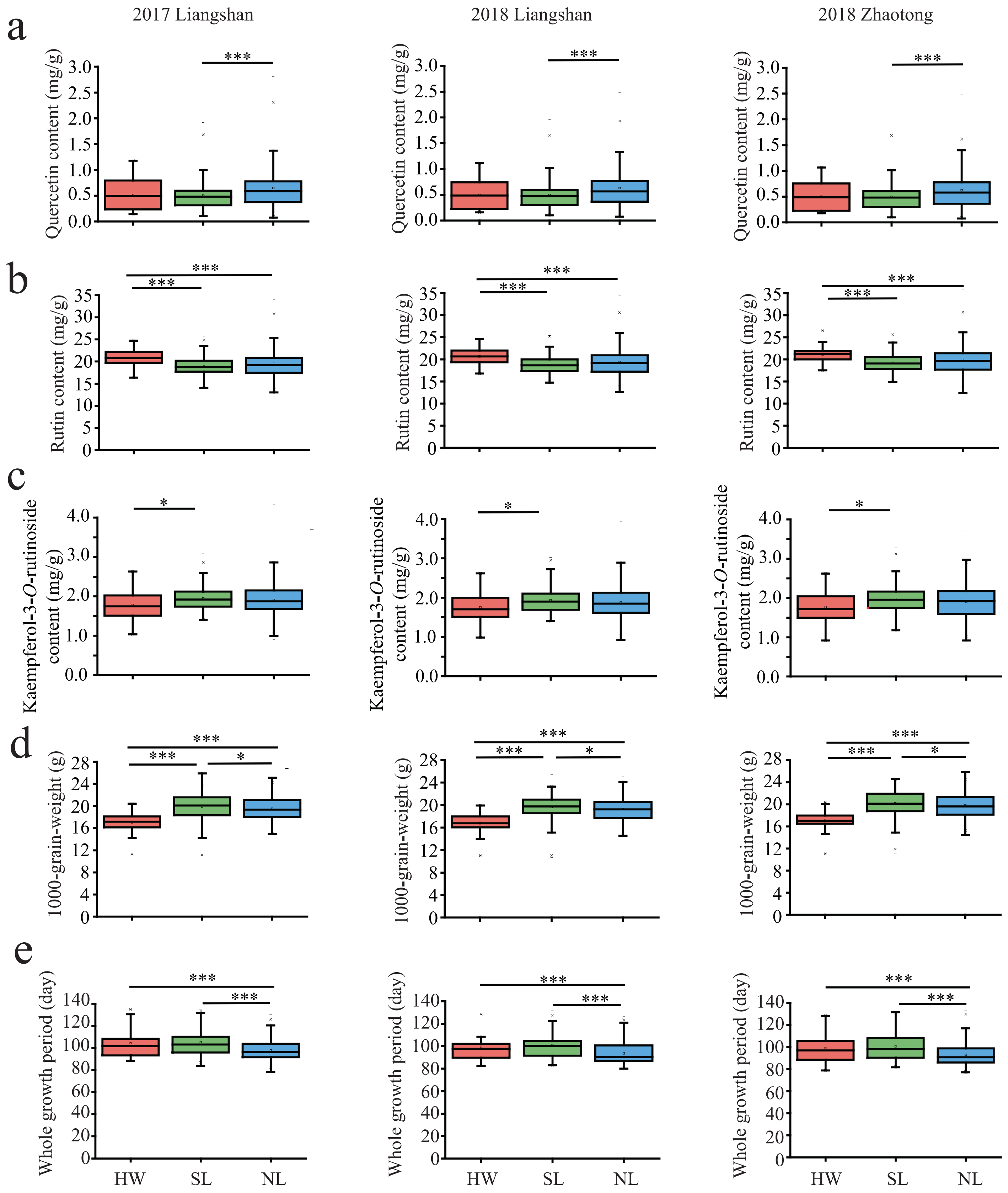

2017 Liangshan
2018 Liangshan
2018 Zhaotong
a
b
c
d
e
Quercetin content (mg/g)
Rutin content (mg/g)
Kaempferol-3-O-rutinoside content (mg/g)
1000-grain-weight (g)
Whole growth period (day)
HW
SL
NL
***
*

Figure S7

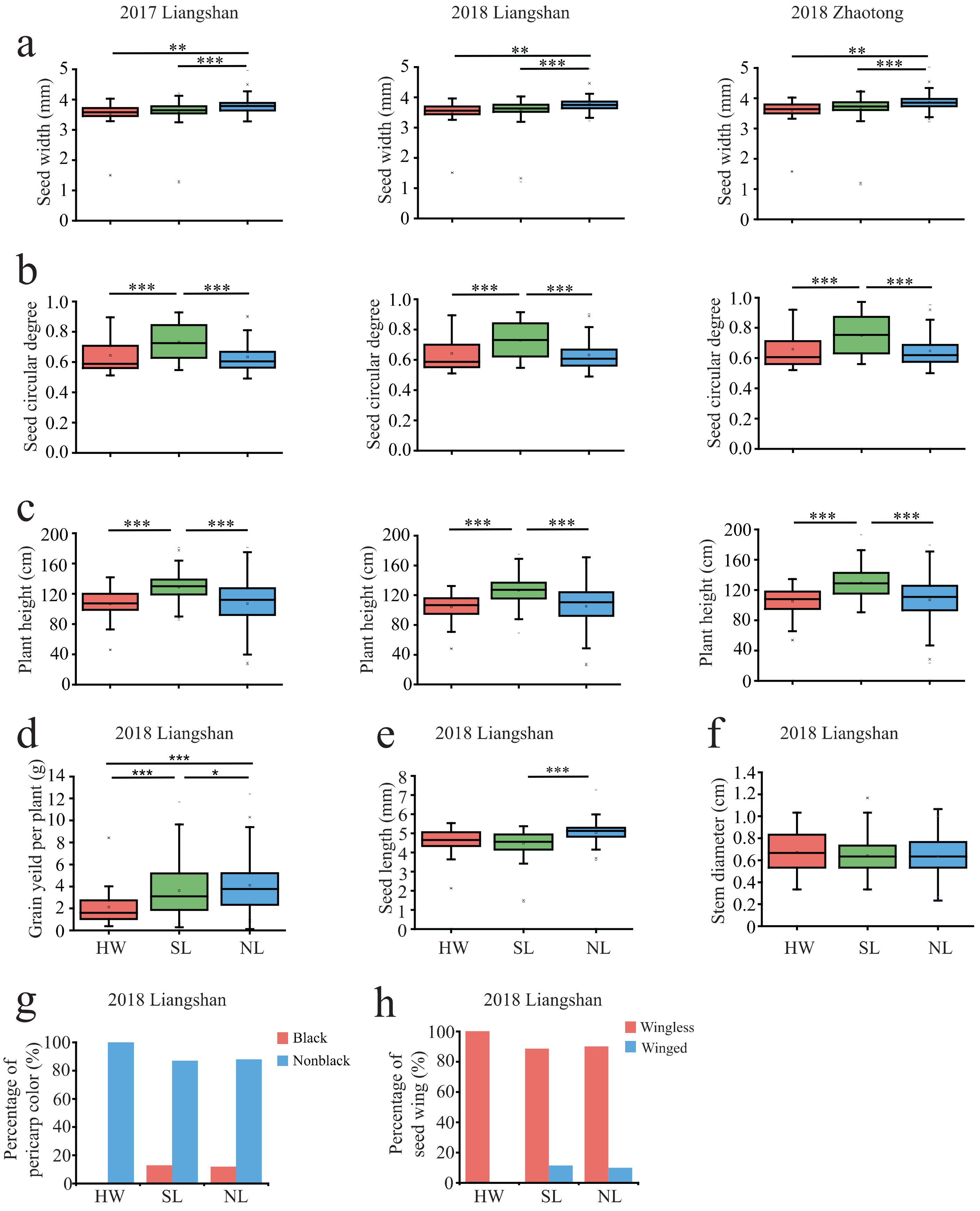
2017 Liangshan
2018 Liangshan
2018 Zhaotong
a
Seed width (mm)
**
***
b
Seed circular degree
***
c
Plant height (cm)
d
2018 Liangshan
Grain yeild per plant (g)
*
e
Seed length (mm)
f
Stem diameter (cm)
HW
SL
NL
g
Percentage of pericarp color (%)
Black
Nonblack
h
Percentage of seed wing (%)
Wingless
Winged

Figure S6 and S7 Quantification of agronomic and quality traits of 480 accessions in three groups. For HW, n = 35; for SL, n = 203; for NL, n = 242. The box plots reflect 25%, 50%, 75%, and the maximum/minimum of the total value. *$P < 0.05$, **$P < 0.01$, ***$P < 0.001$, Student's t-test.

Figure S8

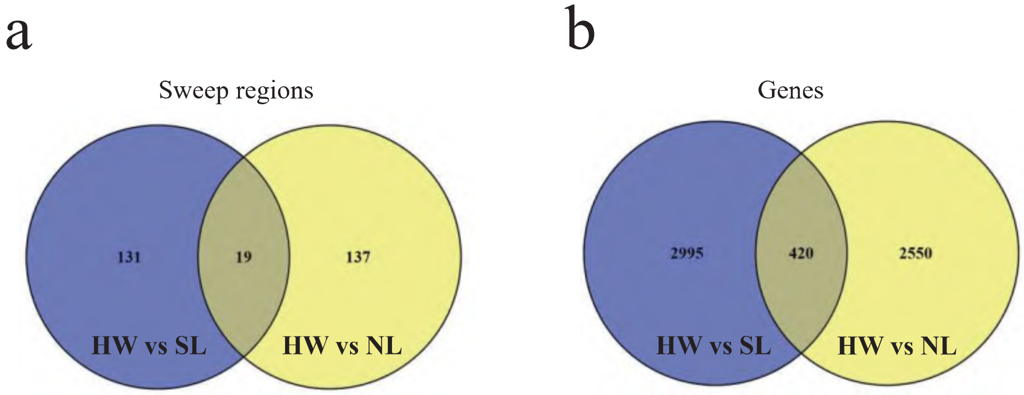
a
Sweep regions
131
19
137
HW vs SL
HW vs NL
b
Genes
2995
420
2550
HW vs SL
HW vs NL

Figure S8 Venn diagram for the overlap of selective sweeps (a) and genes (b) correlated with independent domestication between SL and NL.

Figure S9

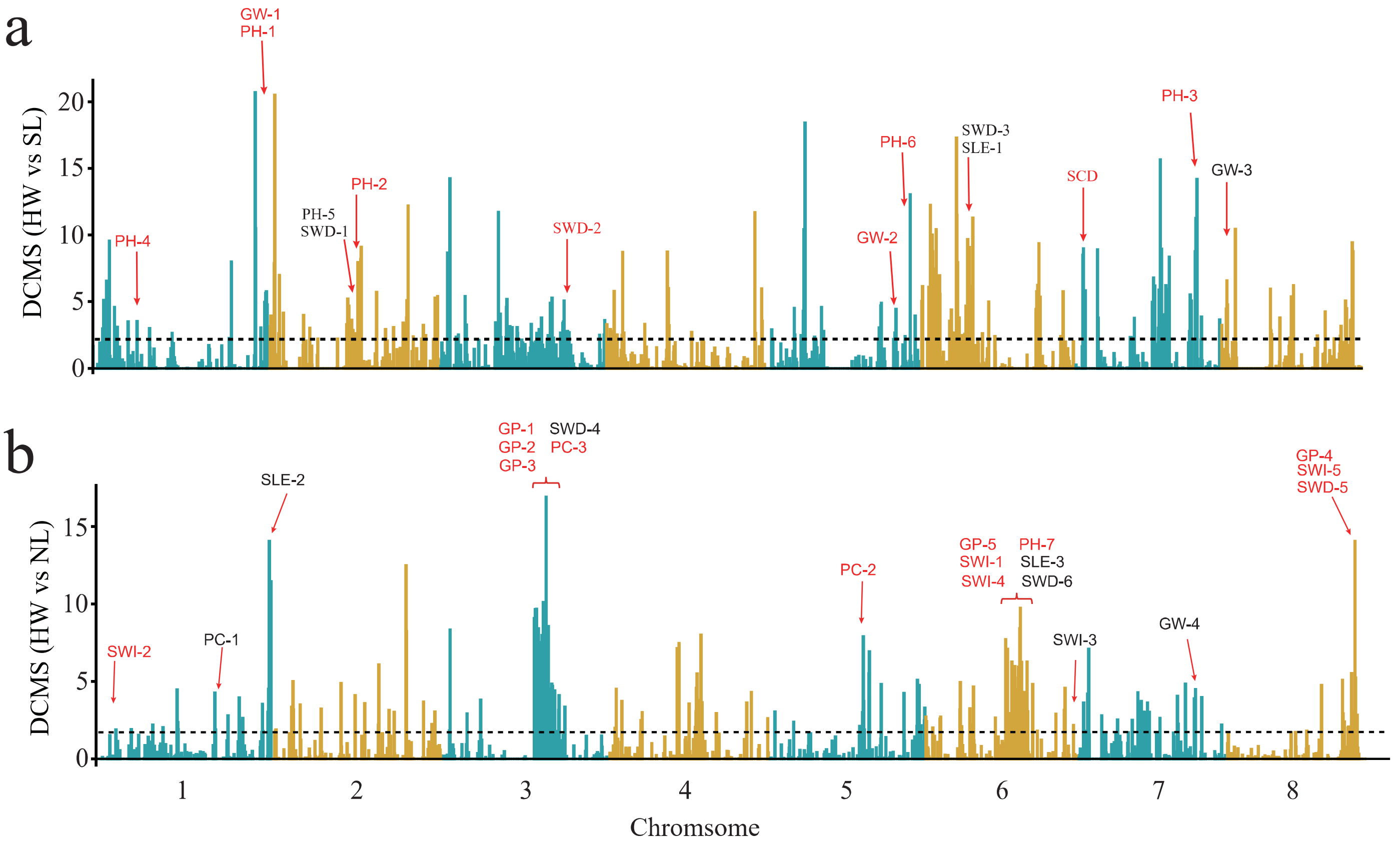

a
GW-1
PH-1
PH-4
PH-5
SWD-1
PH-2
SWD-2
GW-2
PH-6
SWD-3
SLE-1
SCD
PH-3
GW-3
DCMS (HW vs SL)
b
SWI-2
PC-1
SLE-2
GP-1
GP-2
GP-3
SWD-4
PC-3
PC-2
GP-5
SWI-1
SWI-4
PH-7
SLE-3
SWD-6
SWI-3
GW-4
GP-4
SWI-5
SWD-5
DCMS (HW vs NL)
Chromsome

Figure S9 Genome wide analysis of selection sweeps during independent domestication used by de-correlated composite of multiple signals (DCMS). Selective signals associated with domestication in SL (top 10%; DCMS > 2.11) (a) and in NL (top 10%; DCMS > 1.65) (b). Red arrows indicate characterized GWAS loci overlapped with selective sweeps. The red font indicates the sweeps overlapped with those calculated by XP-CLR in Figure 2.

Figure S10

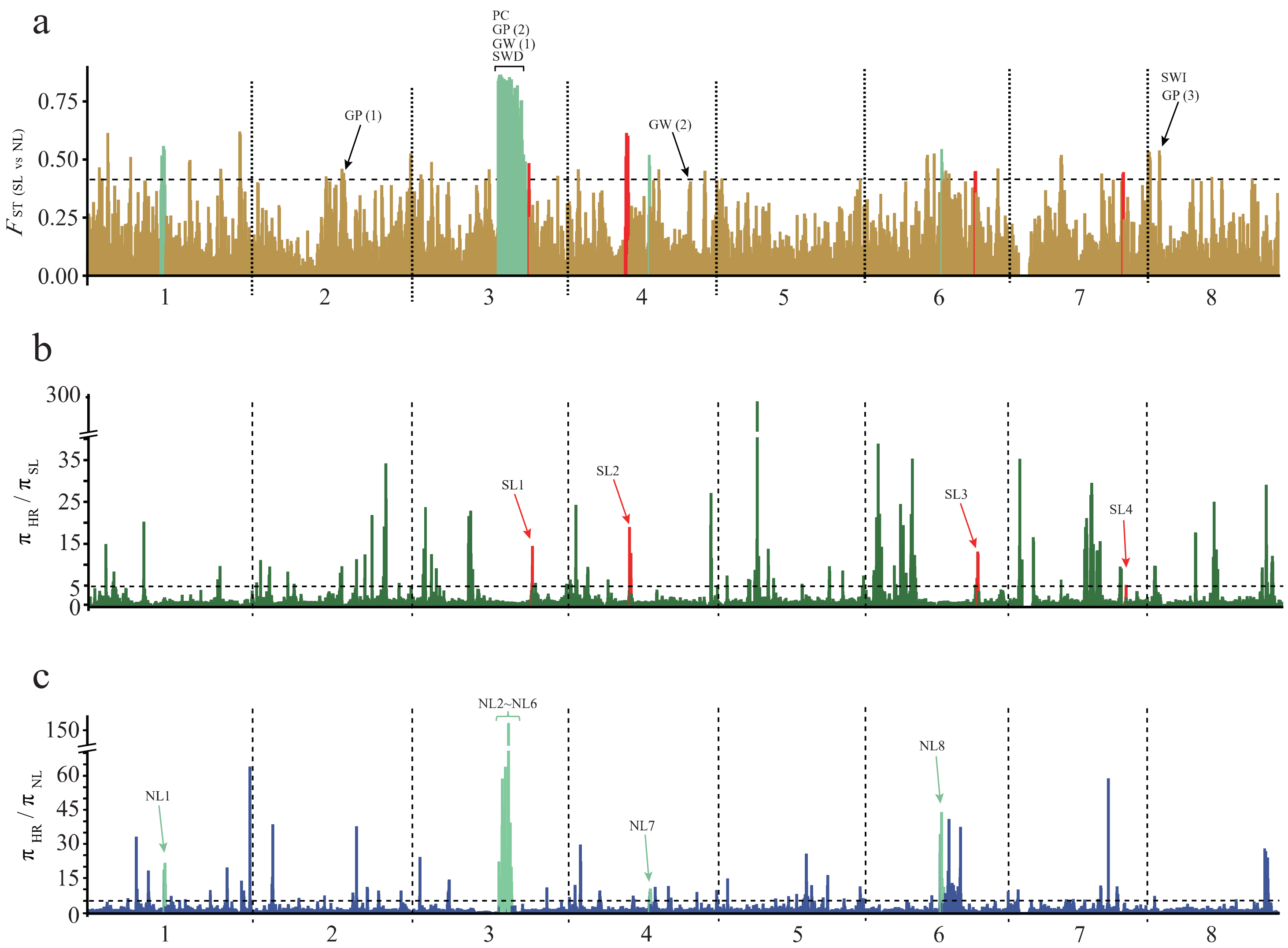

a
PC
GP (2)
GW (1)
SWD
GP (1)
GW (2)
SWI
GP (3)
F_{ST} (SL vs NL)
0.75
0.50
0.25
0.00
1
2
3
4
5
6
7
8
b
300
35
25
15
5
0
π_{HR} / π_{SL}
SL1
SL2
SL3
SL4
c
150
60
45
30
15
0
π_{HR} / π_{NL}
NL2~NL6
NL1
NL7
NL8
1
2
3
4
5
6
7
8

Figure S10 Unique selective sweep regions analysis with F_{ST} and the comparison of nucleotide diversity. a, Whole genome screening of selective sweeps during differentiation between SL and NL. 44 regions (top 5%; $F_{ST} > 0.49$) are found as the candidate differentiation sweeps. Black arrows indicate the overlaps of GWAS loci with sweeps. b,c, 87 (top 5%; $\pi_{HR}/\pi_{SL} > 4.80$) and 80 (top 5%; $\pi_{HR}/\pi_{SL} > 5.12$) selective sweeps are identified by the comparison of whole genome nucleotide diversity between HR and SL (b) and between HR and NL (c). 4 unique selective sweeps in SL and 8 unique selective sweeps in NL are identified by overlapping with the differentiation regions in (a) that showed in red (b) and green bars (c), respectively.

Figure S11

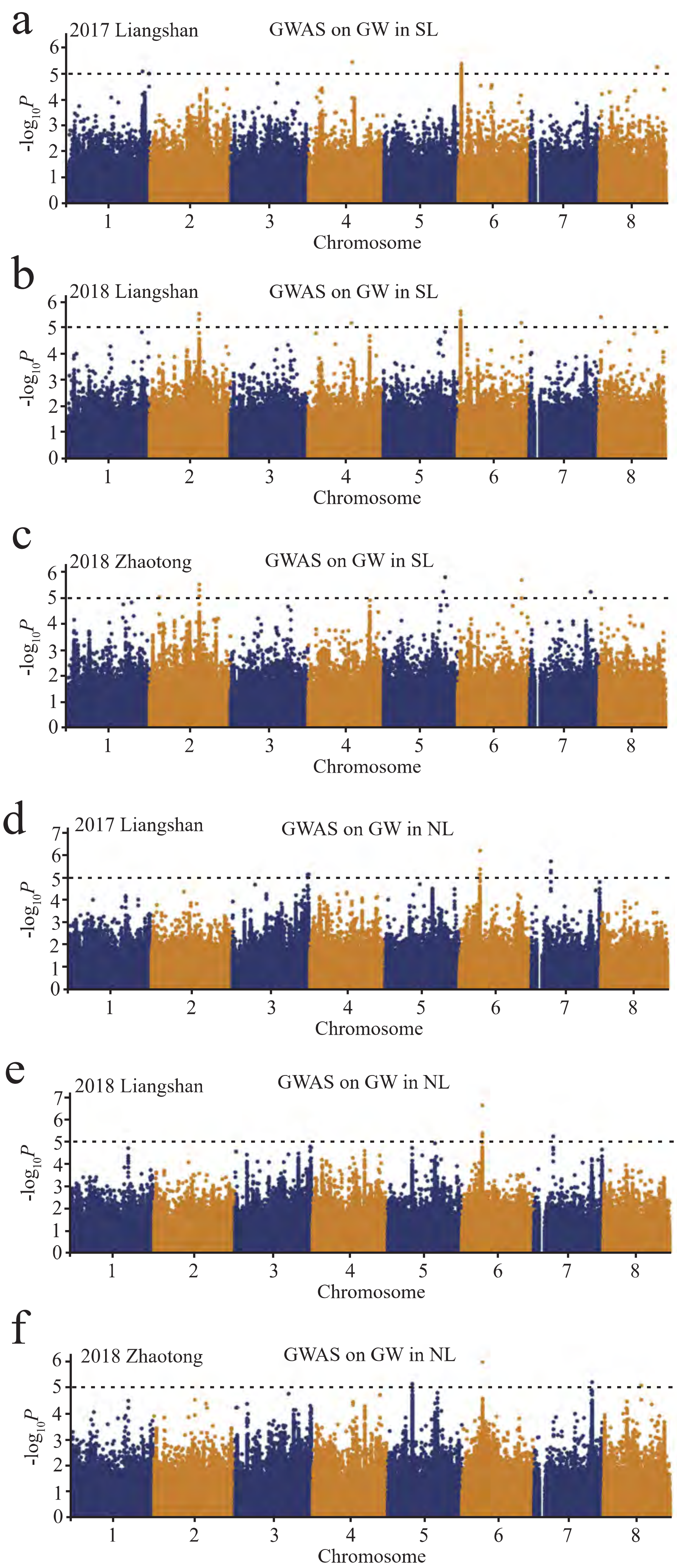
a
2017 Liangshan
GWAS on GW in SL
b
2018 Liangshan
GWAS on GW in SL
c
2018 Zhaotong
GWAS on GW in SL
d
2017 Liangshan
GWAS on GW in NL
e
2018 Liangshan
GWAS on GW in NL
f
2018 Zhaotong
GWAS on GW in NL
$-\log_{10}P$
Chromosome
1 2 3 4 5 6 7 8

Figure S11 GWAS analysis of 1000-grain-weight in SL and NL, respectively. Manhattan plots for GWAS on GW in SL (a-c) and NL (d-e). The dashed line indicates the threshold $-\log P = 5$.

Figure S12

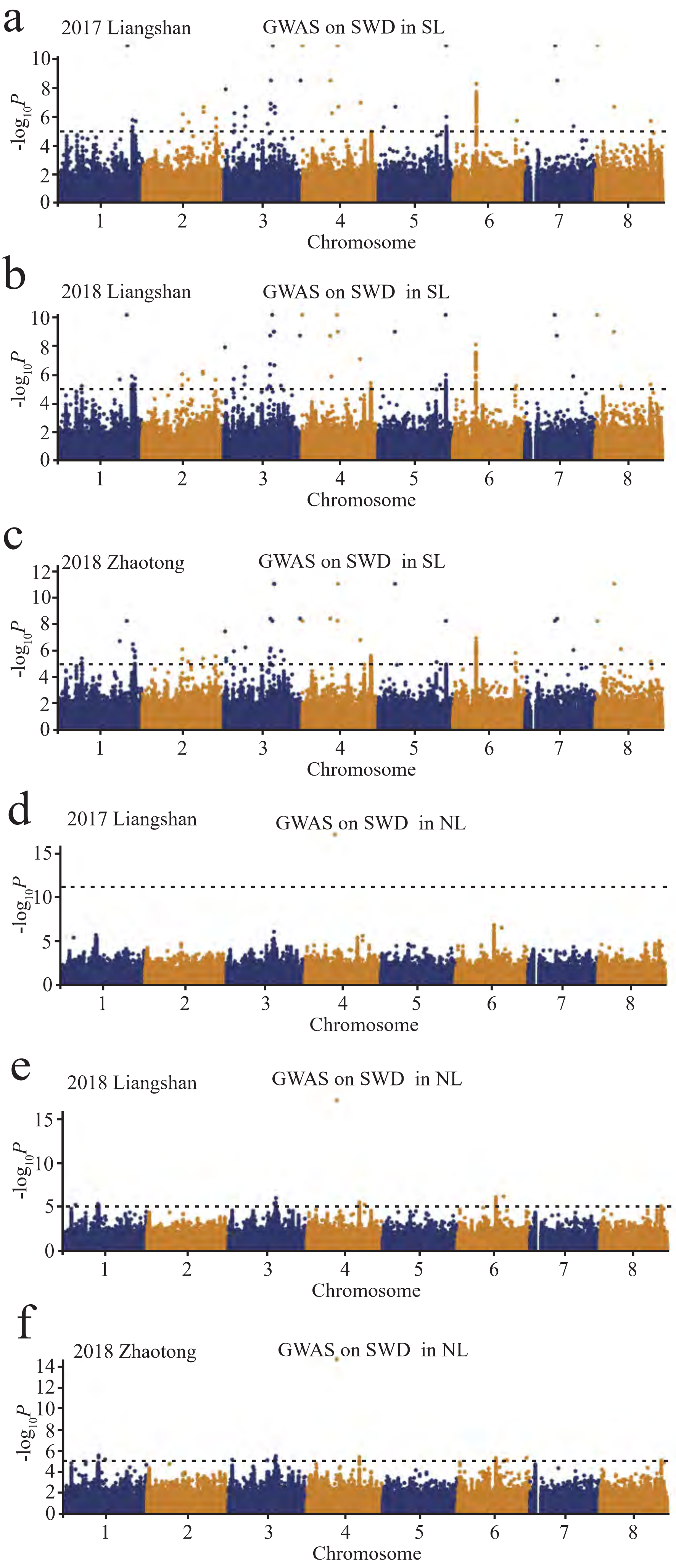

a
2017 Liangshan
GWAS on SWD in SL
$-\log_{10}P$
Chromosome
b
2018 Liangshan
GWAS on SWD in SL
$-\log_{10}P$
Chromosome
c
2018 Zhaotong
GWAS on SWD in SL
$-\log_{10}P$
Chromosome
d
2017 Liangshan
GWAS on SWD in NL
$-\log_{10}P$
Chromosome
e
2018 Liangshan
GWAS on SWD in NL
$-\log_{10}P$
Chromosome
f
2018 Zhaotong
GWAS on SWD in NL
$-\log_{10}P$
Chromosome

Figure S12 GWAS analysis of seed width in SL and NL, respectively. Manhattan plots for GWAS on SWD in SL (a-c) and NL (d-e). The dashed line indicates the threshold -log$P$ = 5.

Figure S13

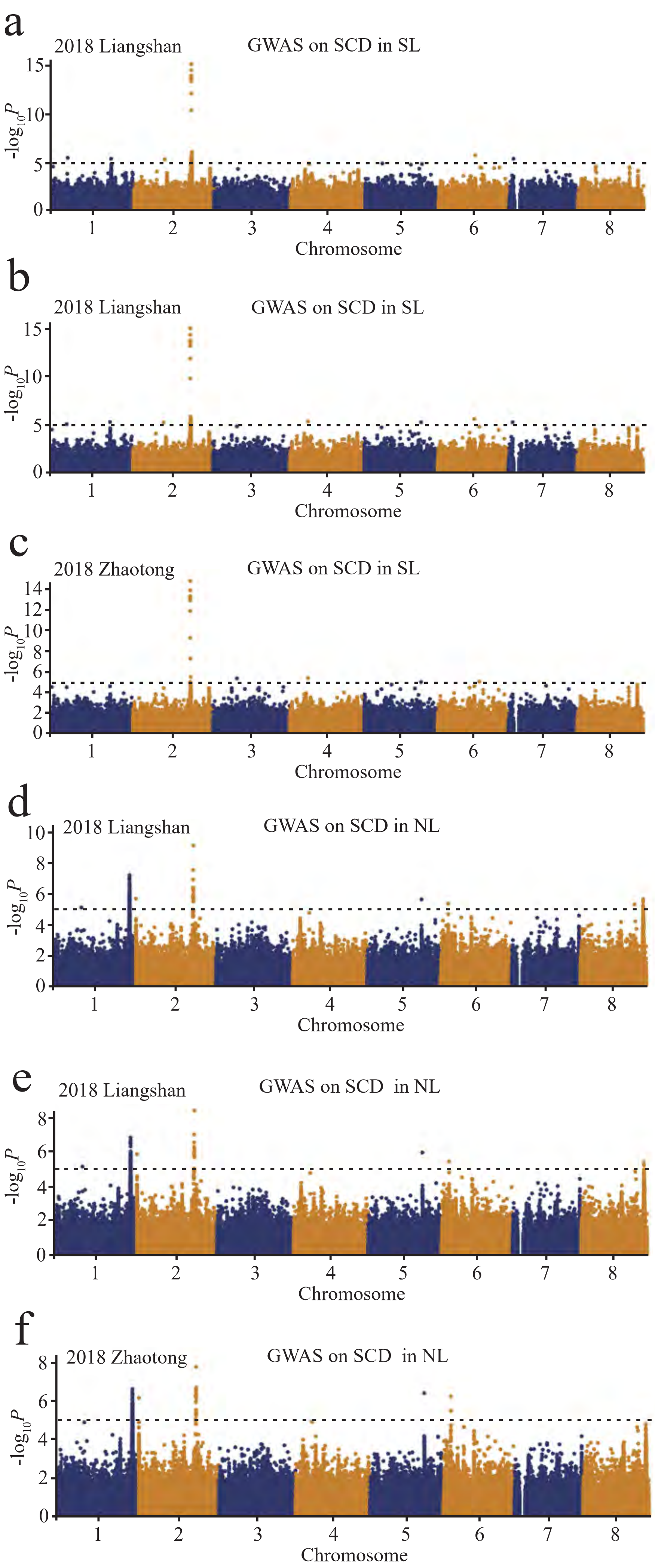
a
2018 Liangshan
GWAS on SCD in SL
-log10P
Chromosome
b
2018 Liangshan
GWAS on SCD in SL
-log10P
Chromosome
c
2018 Zhaotong
GWAS on SCD in SL
-log10P
Chromosome
d
2018 Liangshan
GWAS on SCD in NL
-log10P
Chromosome
e
2018 Liangshan
GWAS on SCD in NL
-log10P
Chromosome
f
2018 Zhaotong
GWAS on SCD in NL
-log10P
Chromosome

Figure S13 GWAS analysis of seed circular degree in SL and NL, respectively. Manhattan plots for GWAS on SCD in SL (a-c) and NL (d-e). The dashed line indicates the threshold -log$P$ = 5.

Figure S14

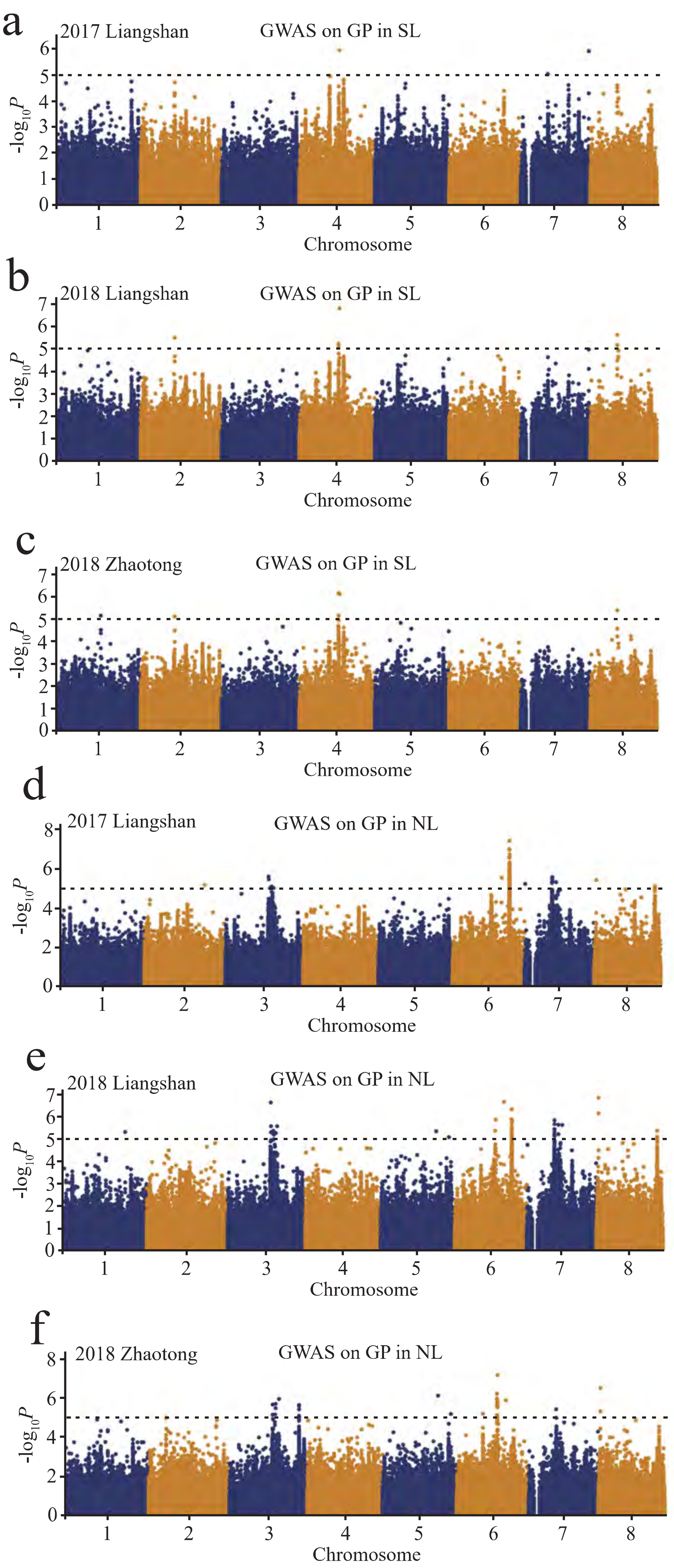
a
2017 Liangshan
GWAS on GP in SL
-log10P
Chromosome
b
2018 Liangshan
GWAS on GP in SL
-log10P
Chromosome
c
2018 Zhaotong
GWAS on GP in SL
-log10P
Chromosome
d
2017 Liangshan
GWAS on GP in NL
-log10P
Chromosome
e
2018 Liangshan
GWAS on GP in NL
-log10P
Chromosome
f
2018 Zhaotong
GWAS on GP in NL
-log10P
Chromosome

Figure S14 GWAS analysis of whole growth period in SL and NL, respectively. Manhattan plots for GWAS on GP in SL (a-c) and NL (d-e). The dashed line indicates the threshold -log$P$ = 5.

Figure S15

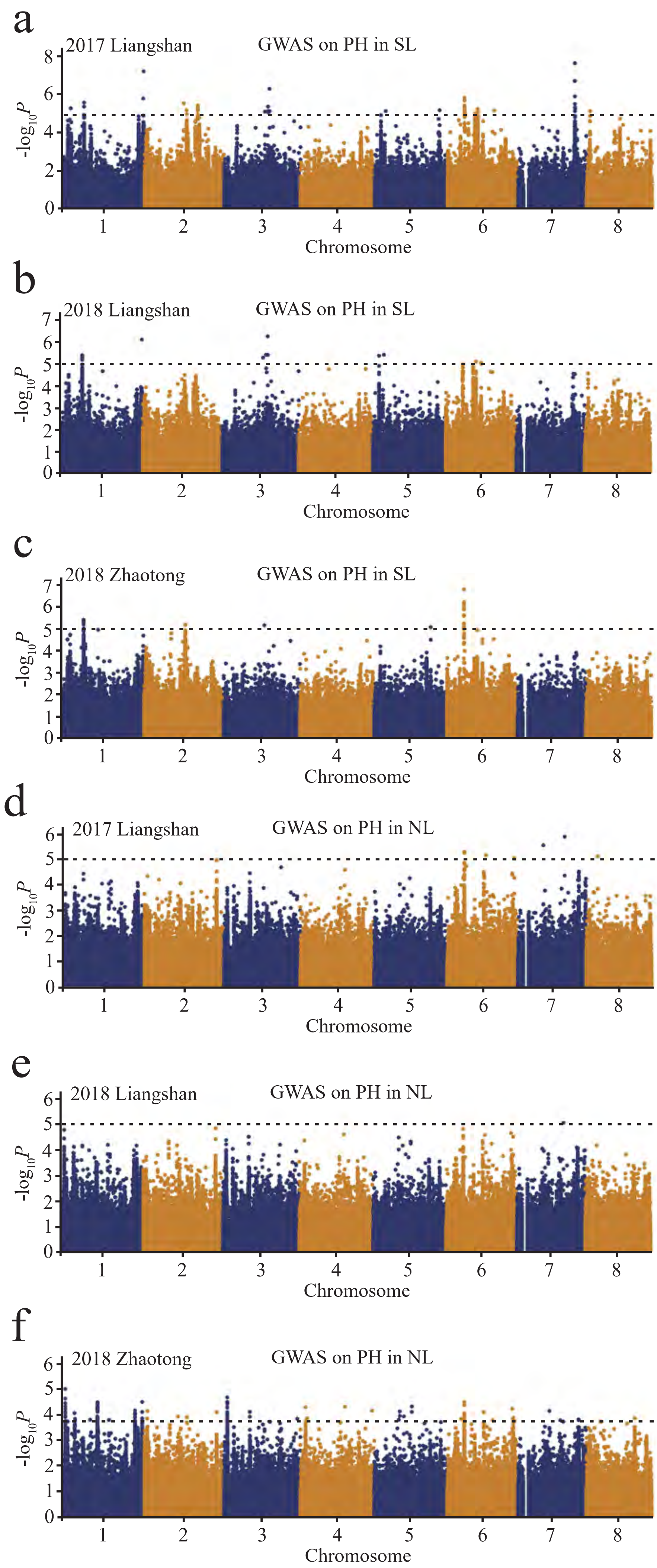
a
2017 Liangshan
GWAS on PH in SL
b
2018 Liangshan
GWAS on PH in SL
c
2018 Zhaotong
GWAS on PH in SL
d
2017 Liangshan
GWAS on PH in NL
e
2018 Liangshan
GWAS on PH in NL
f
2018 Zhaotong
GWAS on PH in NL
-log10P
Chromosome

Figure S15 GWAS analysis of plant height in SL and NL, respectively. Manhattan plots for GWAS on PH in SL (a-c) and NL (d-e). The dashed line indicates the threshold -log$P$ = 5.

Figure S16

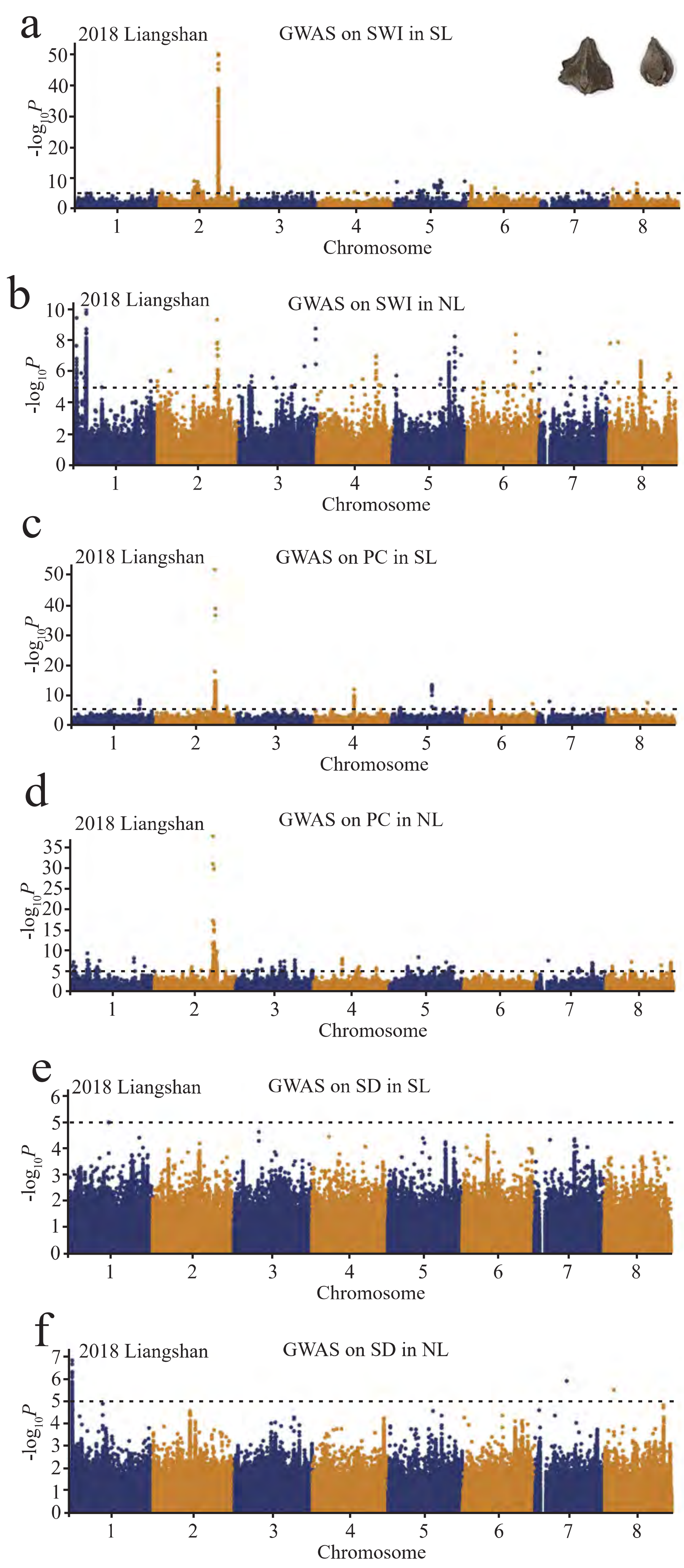
a
2018 Liangshan
GWAS on SWI in SL
b
2018 Liangshan
GWAS on SWI in NL
c
2018 Liangshan
GWAS on PC in SL
d
2018 Liangshan
GWAS on PC in NL
e
2018 Liangshan
GWAS on SD in SL
f
2018 Liangshan
GWAS on SD in NL
$-\log_{10}P$
Chromosome
1
2
3
4
5
6
7
8

Figure S16 GWAS analysis of agronomic traits in SL and NL, respectively. Manhattan plots for GWAS on SWI (a, b), PC (c, d) and SD (e, f). The dashed line indicates the threshold -log*P* = 5. SWI, seed wing; PC, pericarp color; SD, seed diameter.

Figure S17

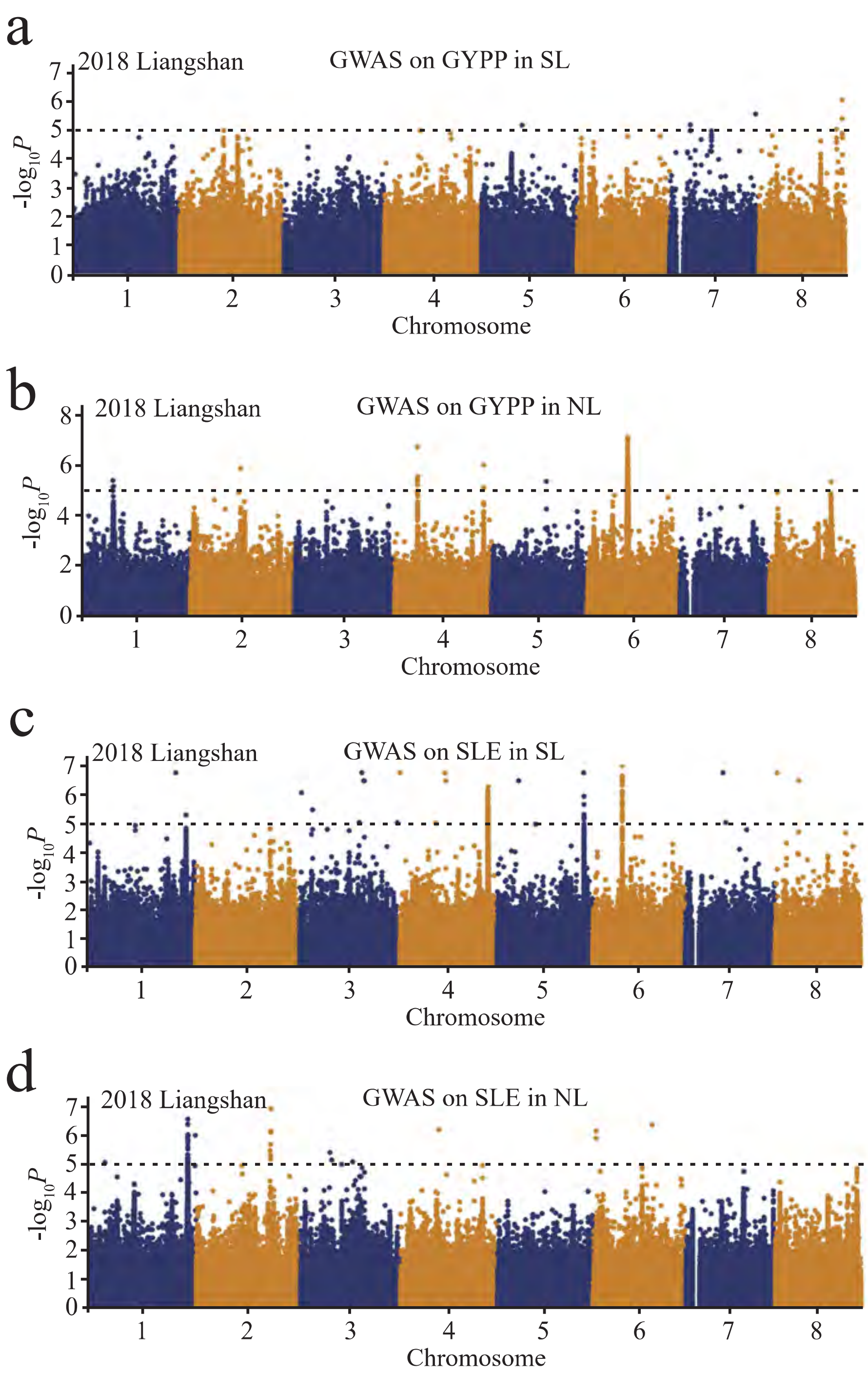
a
2018 Liangshan
GWAS on GYPP in SL
-log10P
Chromosome
b
2018 Liangshan
GWAS on GYPP in NL
-log10P
Chromosome
c
2018 Liangshan
GWAS on SLE in SL
-log10P
Chromosome
d
2018 Liangshan
GWAS on SLE in NL
-log10P
Chromosome

Figure S17 GWAS analysis of agronomic traits in SL and NL, respectively. Manhattan plots for GWAS on GYPP (a, b) and SLE (c, d). The dashed line indicates the threshold -log$P$ = 5. GYPP, grain yield per plant; SLE, seed length.

Figure S18

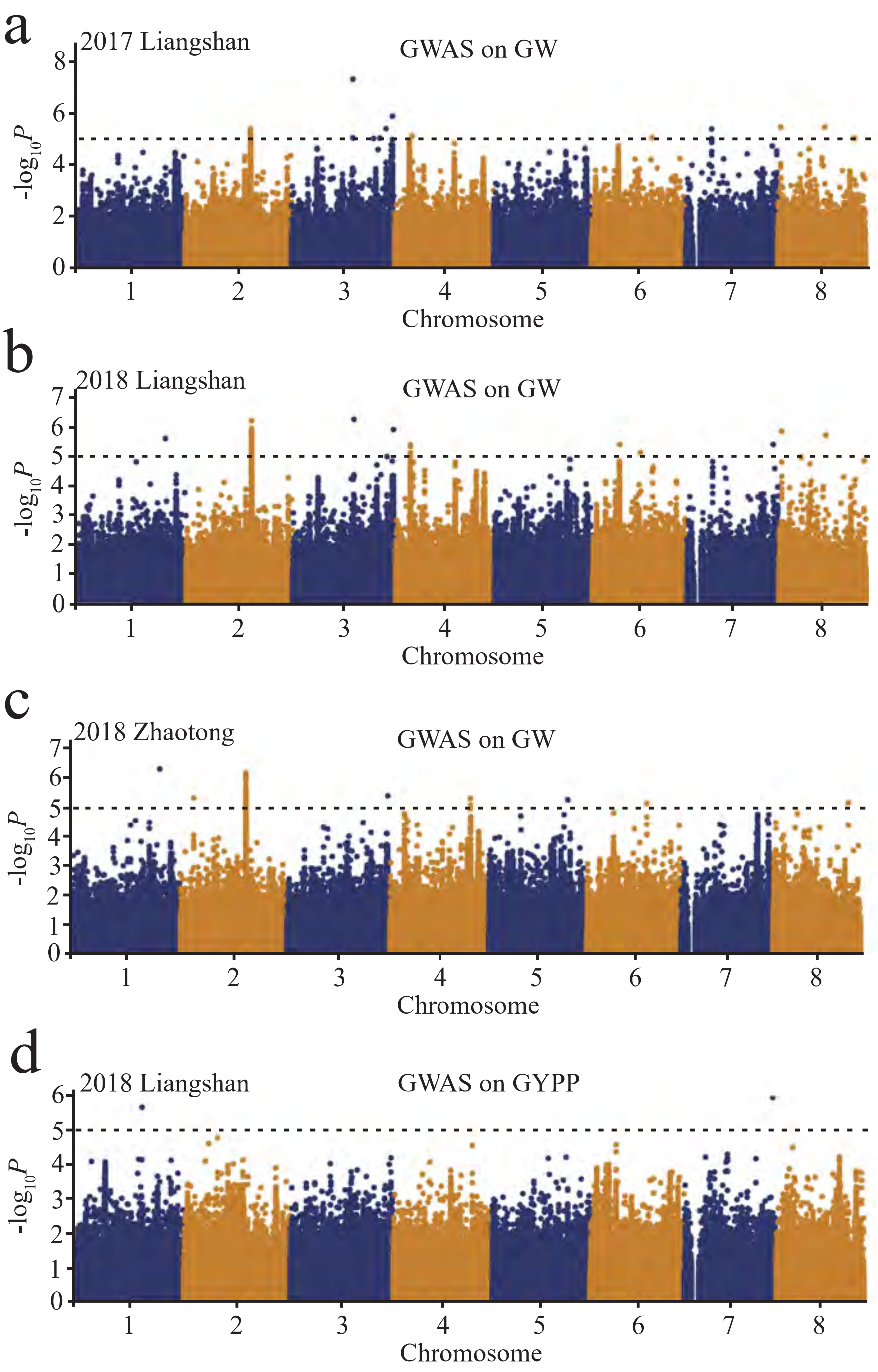

a
2017 Liangshan
GWAS on GW
-log10P
Chromosome
b
2018 Liangshan
GWAS on GW
-log10P
Chromosome
c
2018 Zhaotong
GWAS on GW
-log10P
Chromosome
d
2018 Liangshan
GWAS on GYPP
-log10P
Chromosome

Figure S18 GWAS analysis of differentiation traits. Manhattan plots for GWAS on GW (a-c) and GYPP (d). The dashed line indicates the threshold -log$P$ = 5. GW, 1000-grain-weight; GYPP, grain yield per plant.

Figure S19

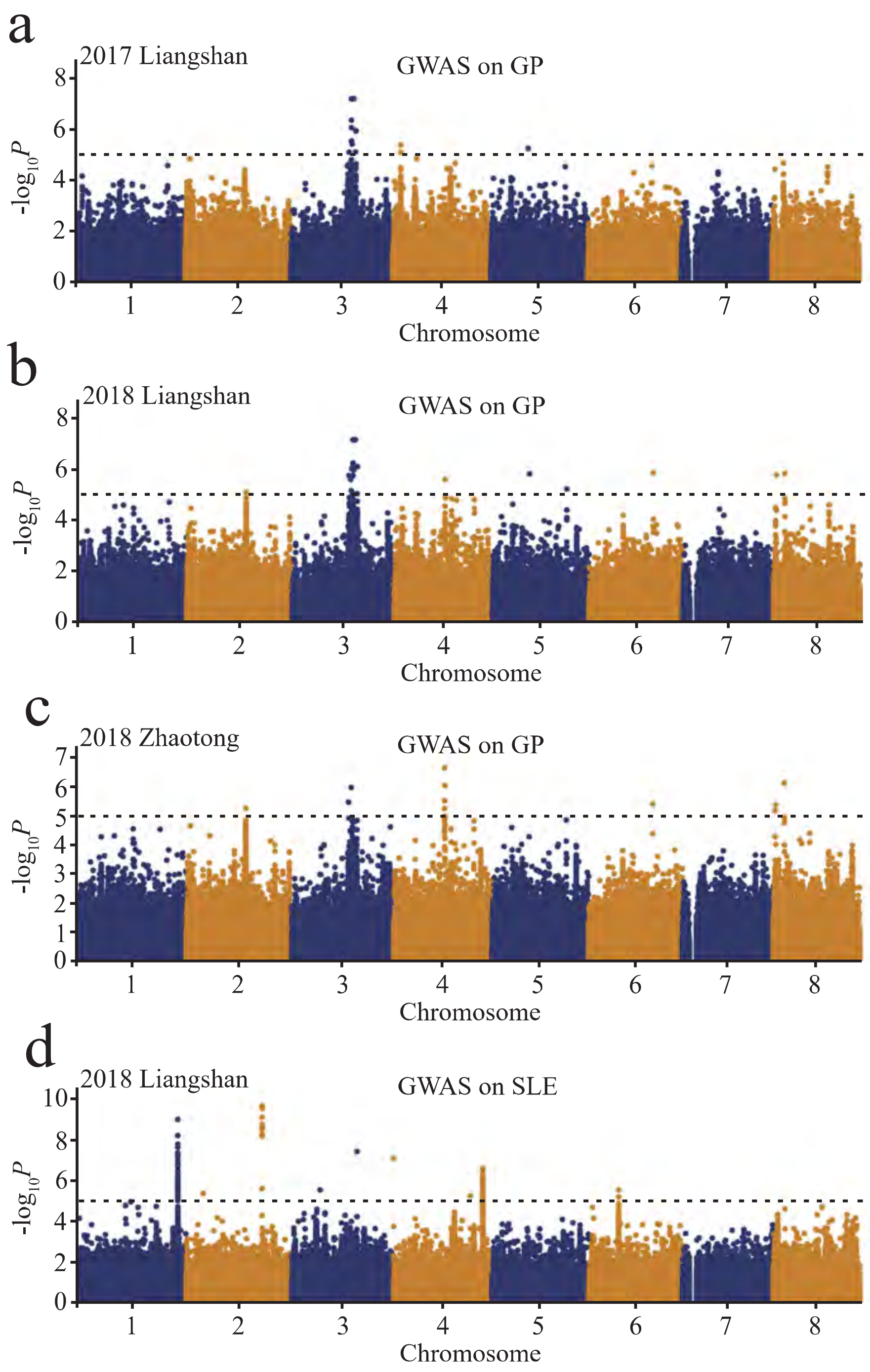
a
2017 Liangshan
GWAS on GP
-log10P
Chromosome
b
2018 Liangshan
GWAS on GP
-log10P
Chromosome
c
2018 Zhaotong
GWAS on GP
-log10P
Chromosome
d
2018 Liangshan
GWAS on SLE
-log10P
Chromosome

Figure S19 GWAS analysis of differentiation traits. Manhattan plots for GWAS on GP (a-c) and SLE (d). The dashed line indicates the threshold -log$P$ = 5. GP, whole grow period; SLE, seed length.

Figure S20

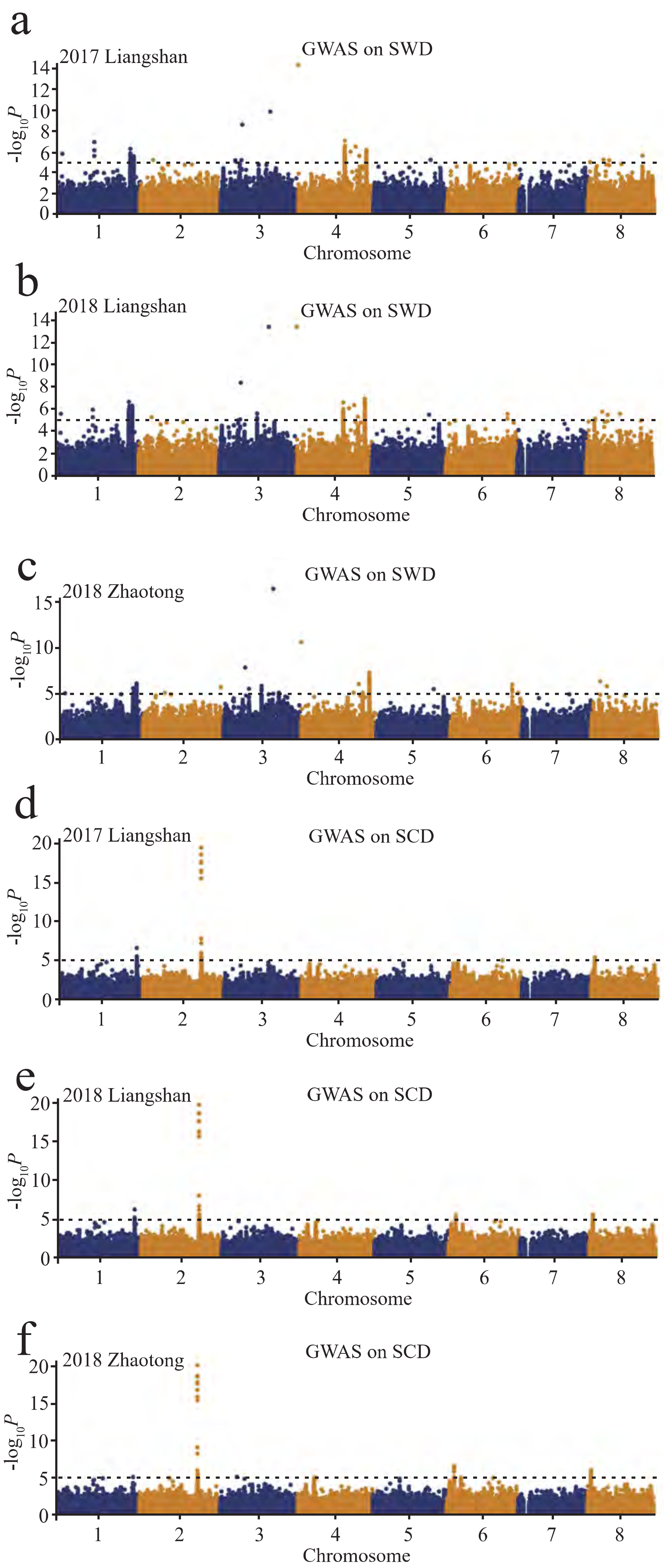

a
2017 Liangshan
GWAS on SWD
-log10P
Chromosome
b
2018 Liangshan
GWAS on SWD
-log10P
Chromosome
c
2018 Zhaotong
GWAS on SWD
-log10P
Chromosome
d
2017 Liangshan
GWAS on SCD
-log10P
Chromosome
e
2018 Liangshan
GWAS on SCD
-log10P
Chromosome
f
2018 Zhaotong
GWAS on SCD
-log10P
Chromosome

Figure S20 GWAS analysis of differentiation traits. Manhattan plots for GWAS on SWD (a-c) and SCD (d-f). The dashed line indicates the threshold -log$P$ = 5. SWD, seed width; SCD, seed circular degree.

Figure S21

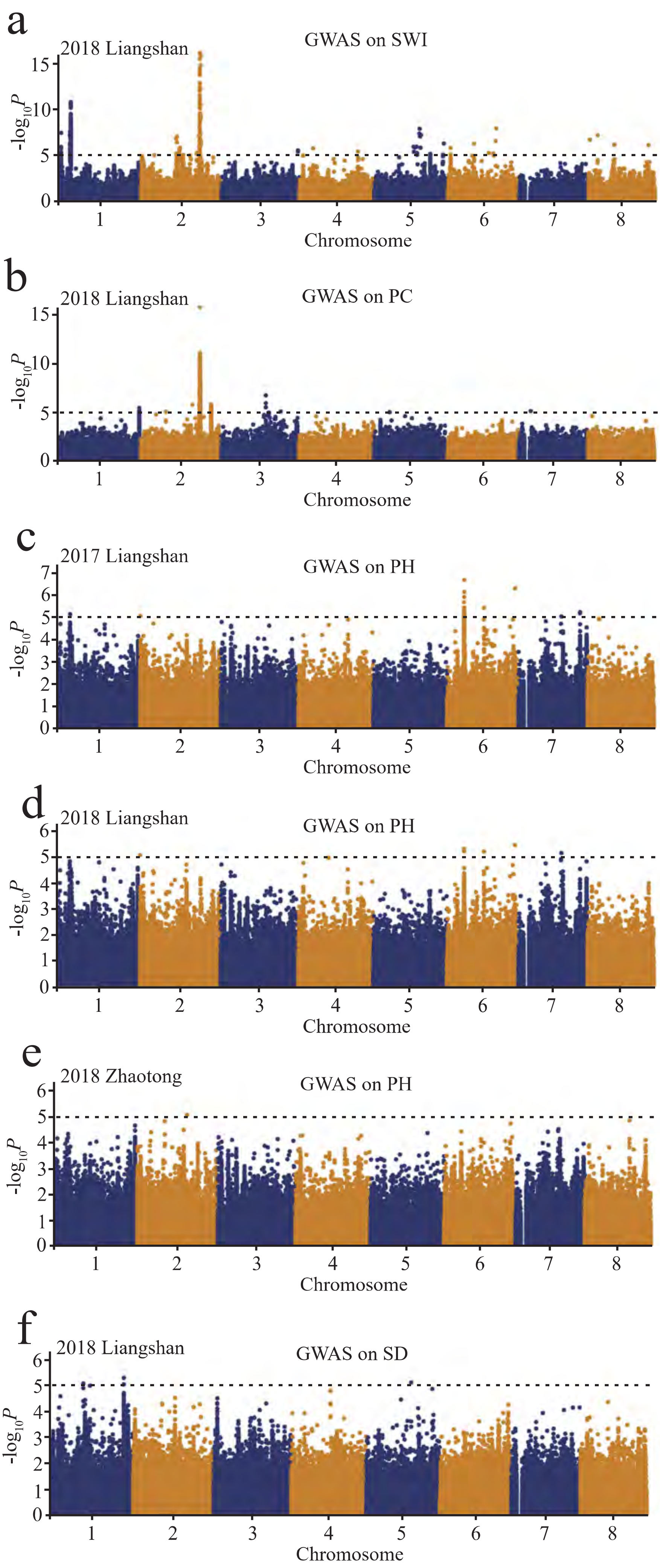
a
2018 Liangshan
GWAS on SWI
$-\log_{10}P$
Chromosome
b
2018 Liangshan
GWAS on PC
$-\log_{10}P$
Chromosome
c
2017 Liangshan
GWAS on PH
$-\log_{10}P$
Chromosome
d
2018 Liangshan
GWAS on PH
$-\log_{10}P$
Chromosome
e
2018 Zhaotong
GWAS on PH
$-\log_{10}P$
Chromosome
f
2018 Liangshan
GWAS on SD
$-\log_{10}P$
Chromosome

Figure S21 GWAS analysis of differentiation traits. Manhattan plots for GWAS on SWI (a), PC (b), PH (c-e) and SD (f). The dashed line indicates the threshold -log*P* = 5. SD, seed diameter. SWI, seed wing; PC, pericarp color; PH, plant height; SD, seed diameter.

Figure S22

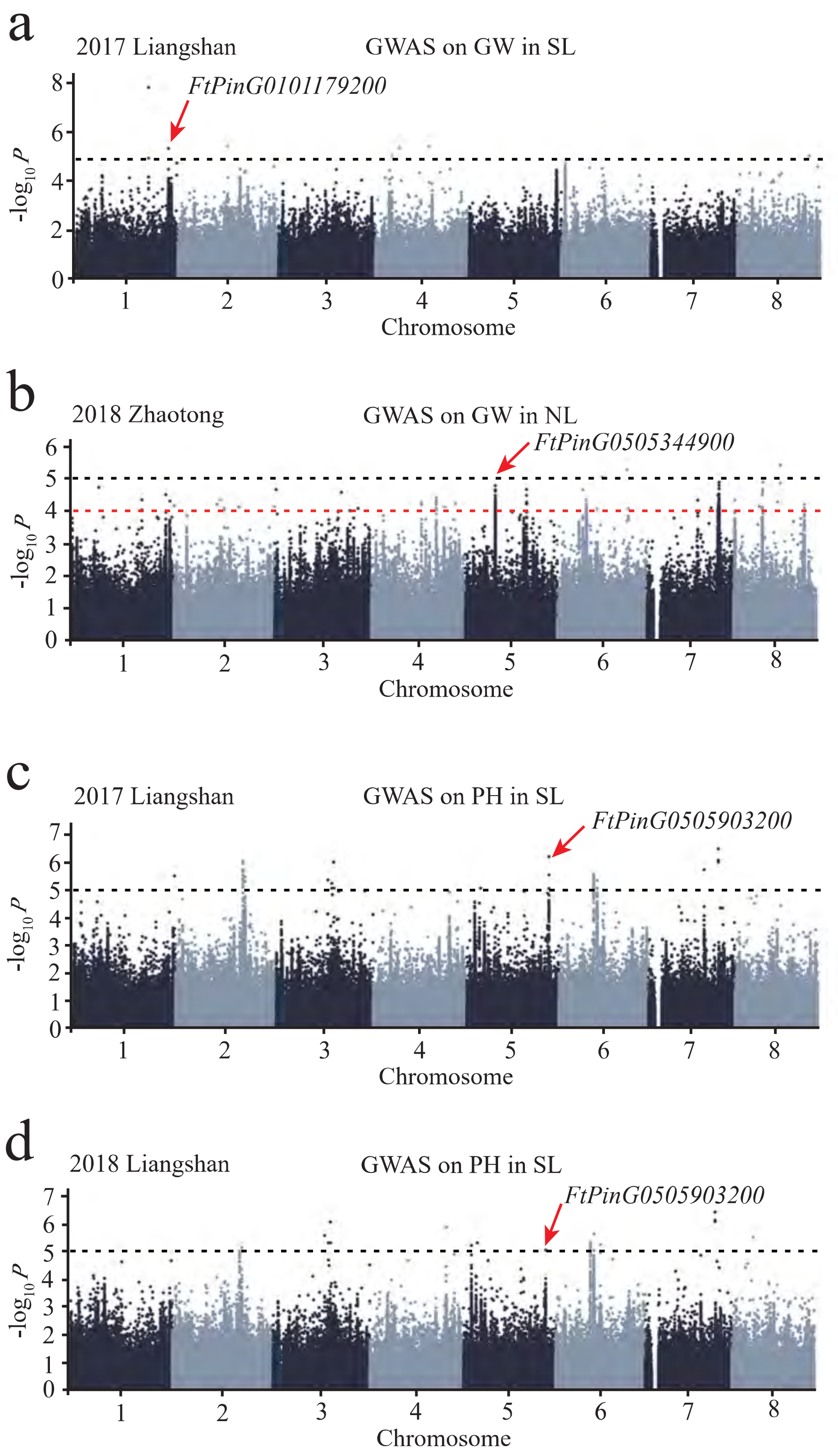
a
2017 Liangshan
GWAS on GW in SL
FtPinG0101179200
-log10 P
Chromosome
b
2018 Zhaotong
GWAS on GW in NL
FtPinG0505344900
-log10 P
Chromosome
c
2017 Liangshan
GWAS on PH in SL
FtPinG0505903200
-log10 P
Chromosome
d
2018 Liangshan
GWAS on PH in SL
FtPinG0505903200
-log10 P
Chromosome

Figure S22 GWAS analysis of GW and PH in SL and NL used by FaST-LMM, respectively. Manhattan plots for GWAS on GW in SL and NL (a, b), and PH in SL (c, d). Candidate genes are indicated by red arrows. The black dashed line indicates the threshold -log$P$ = 5 and the red one indicates -log$P$ = 4.

Figure S23

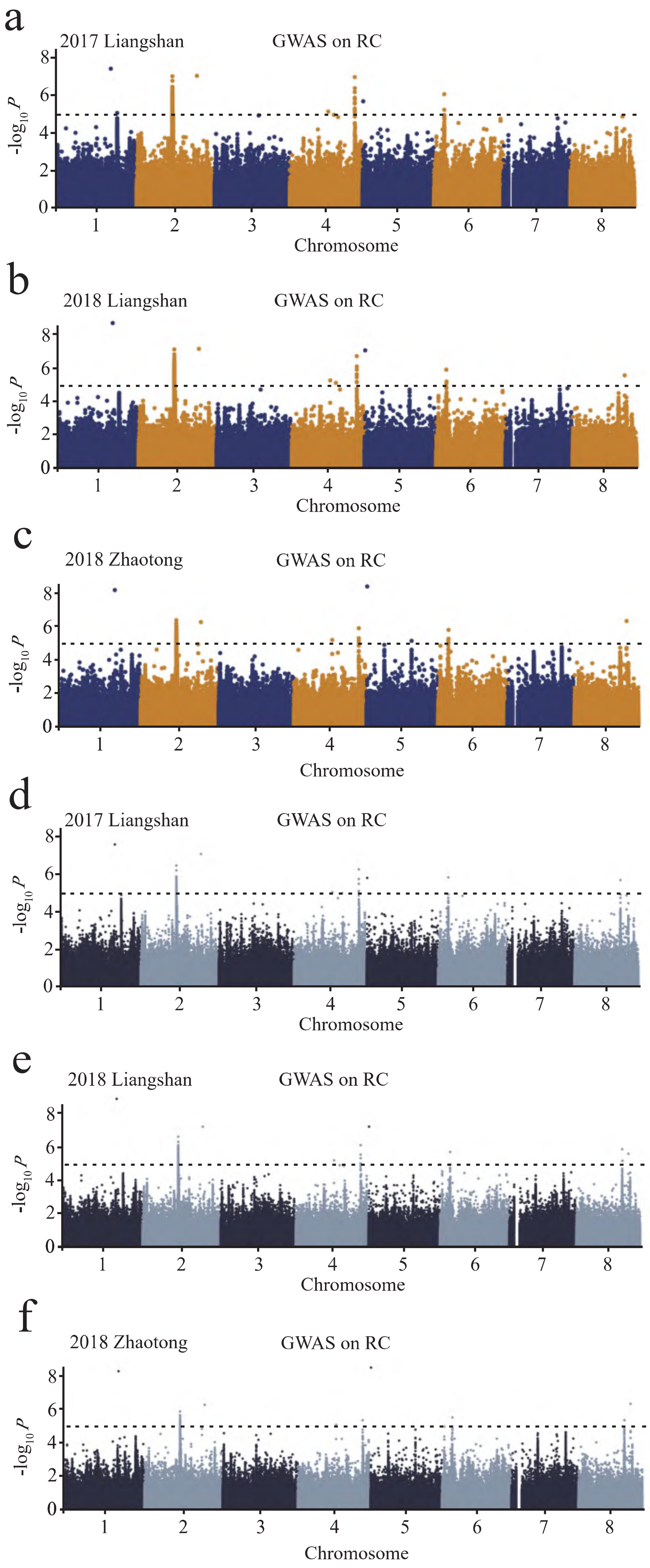
a
2017 Liangshan
GWAS on RC
b
2018 Liangshan
GWAS on RC
c
2018 Zhaotong
GWAS on RC
d
2017 Liangshan
GWAS on RC
e
2018 Liangshan
GWAS on RC
f
2018 Zhaotong
GWAS on RC
$-\log_{10}P$
Chromosome
1
2
3
4
5
6
7
8

Figure S23 GWAS analysis of rutin content. a-c, Manhattan plots for GWAS are calculated by EMMAx. d-f, Manhattan plots are calculated by FaST-LMM. The dashed line indicates the threshold -log*P* = 5.

Figure S24

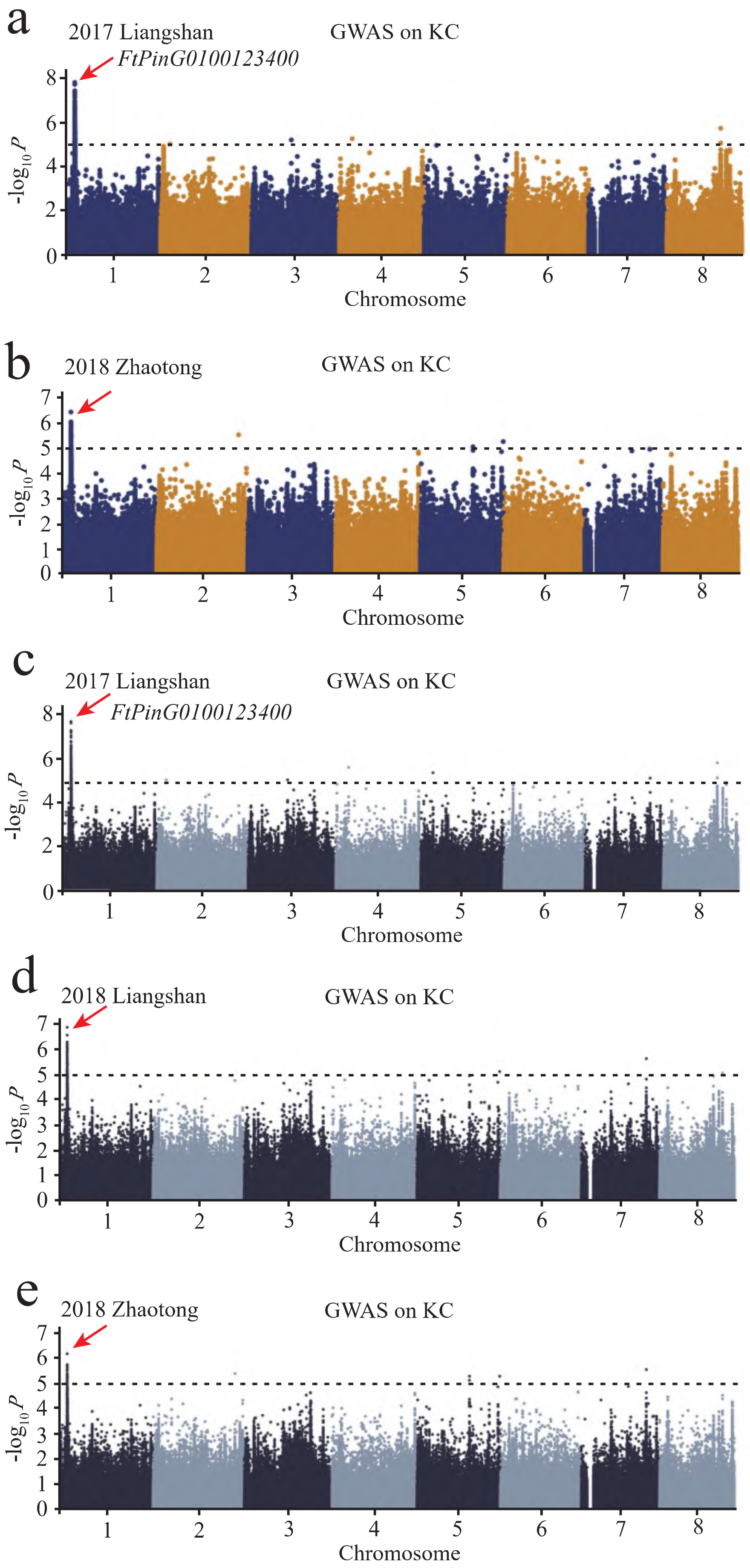
a
2017 Liangshan
GWAS on KC
FtPinG0100123400
-log10P
Chromosome
b
2018 Zhaotong
GWAS on KC
-log10P
Chromosome
c
2017 Liangshan
GWAS on KC
FtPinG0100123400
-log10P
Chromosome
d
2018 Liangshan
GWAS on KC
-log10P
Chromosome
e
2018 Zhaotong
GWAS on KC
-log10P
Chromosome

Figure S24 GWAS analysis of kaempferol-3-*O*-rutinoside content. a-c, Manhattan plots for GWAS are calculated by EMMAx. d-f, Manhattan plots are calculated by FaST-LMM. Red arrows indicate the candidate gene. The dashed line indicates the threshold $-\log P = 5$.

Figure S25

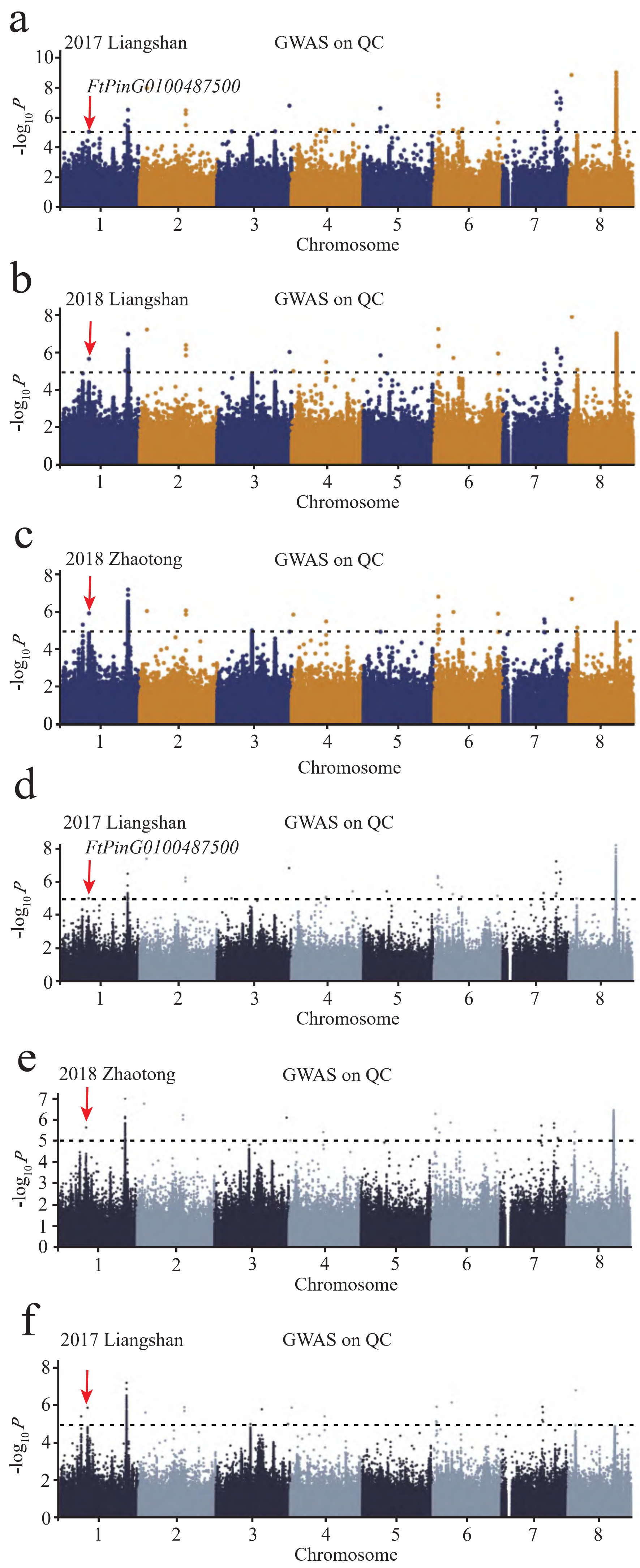
a
2017 Liangshan
GWAS on QC
FtPinG0100487500
$-\log_{10}P$
Chromosome
b
2018 Liangshan
GWAS on QC
c
2018 Zhaotong
GWAS on QC
d
2017 Liangshan
GWAS on QC
FtPinG0100487500
e
2018 Zhaotong
GWAS on QC
f
2017 Liangshan
GWAS on QC

Figure S25 GWAS analysis of quercetin content. a-c, Manhattan plots for GWAS are calculated by EMMAx. d-f, Manhattan plots are calculated by FaST-LMM. Red arrows indicate the candidate gene. The dashed line indicates the threshold -log*P* = 5.

Figure S26

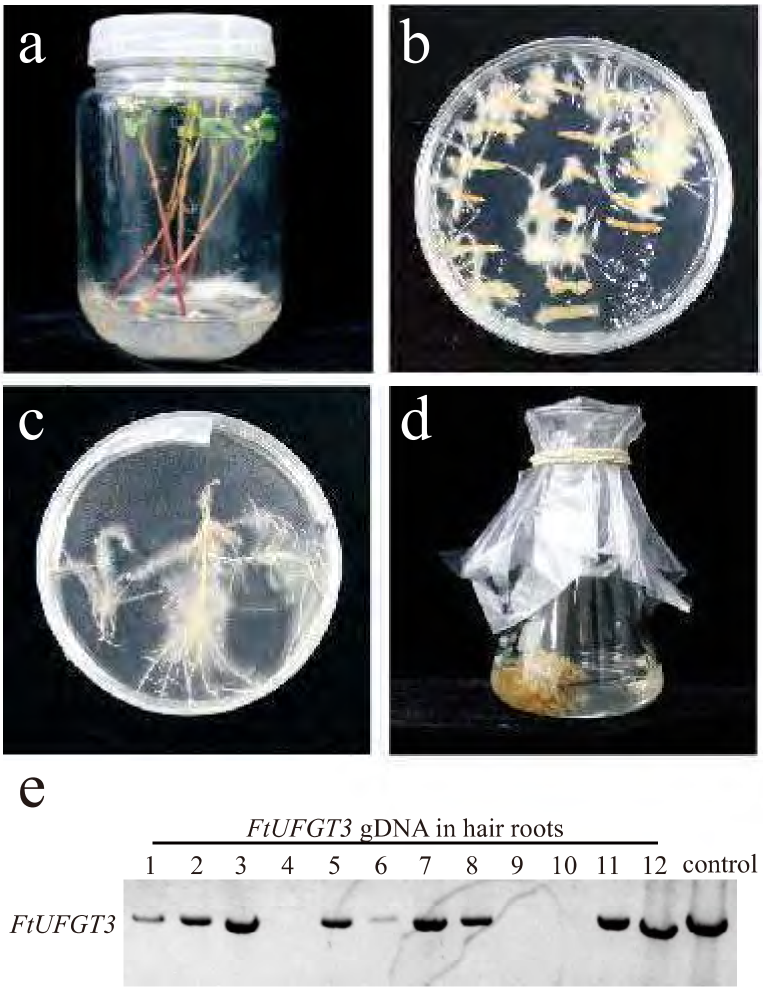
a
b
c
d
e
FtUFGT3 gDNA in hair roots
1 2 3 4 5 6 7 8 9 10 11 12 control
FtUFGT3

Figure S26 Hair root transgenic system in buckwheat. a, Ten-day-old buckwheat seedlings grown on MS media. b, Four-day-old explants of buckwheat after transformation by *Agrobacterium rhizogenes* solution carrying the target vector. c, Seven-day-old single hair root line selected from explants after five-day-old culture. d, One-month-old hairy root in liquid medium transferred from (c). e, PCR-based identification of positive transgenic hairy roots. Primers used in this test are listed in Table S24.

Figure S27

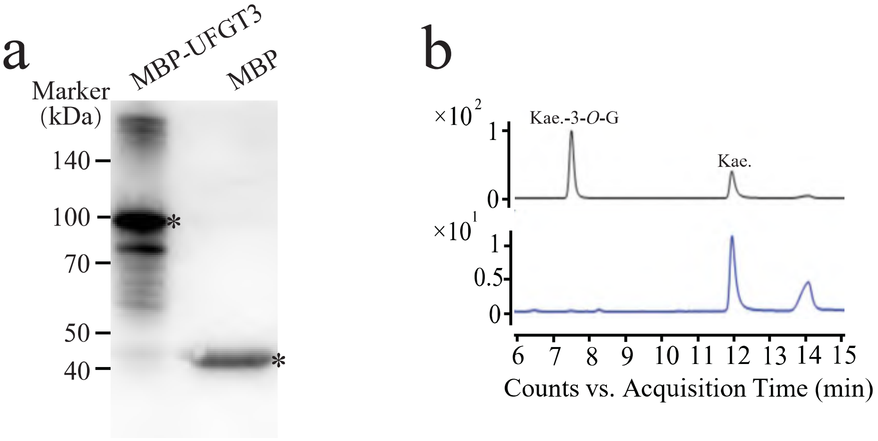
a
Marker (kDa)
MBP-UFGT3
MBP
140
100
70
50
40
*
*
b
×10^2
Kae.-3-O-G
Kae.
1
0
×10^1
1
0.5
0
6 7 8 9 10 11 12 13 14 15
Counts vs. Acquisition Time (min)

Figure S27 *In vitro* enzyme assay of FtUFGT3. a, Purification of recombinant MBP-FtUFGT3 and MBP Protein. The target bands are marked by *. b, Controls of FtUFGT3 substrate analysis *in vitro* by LC/MS in Figure 3i. Up for standers of kaempferol and kaempferol-3-*O*-glucoside; below for reaction product of MBP protein and kaempferol. Kae., kaempferol.

Figure S28

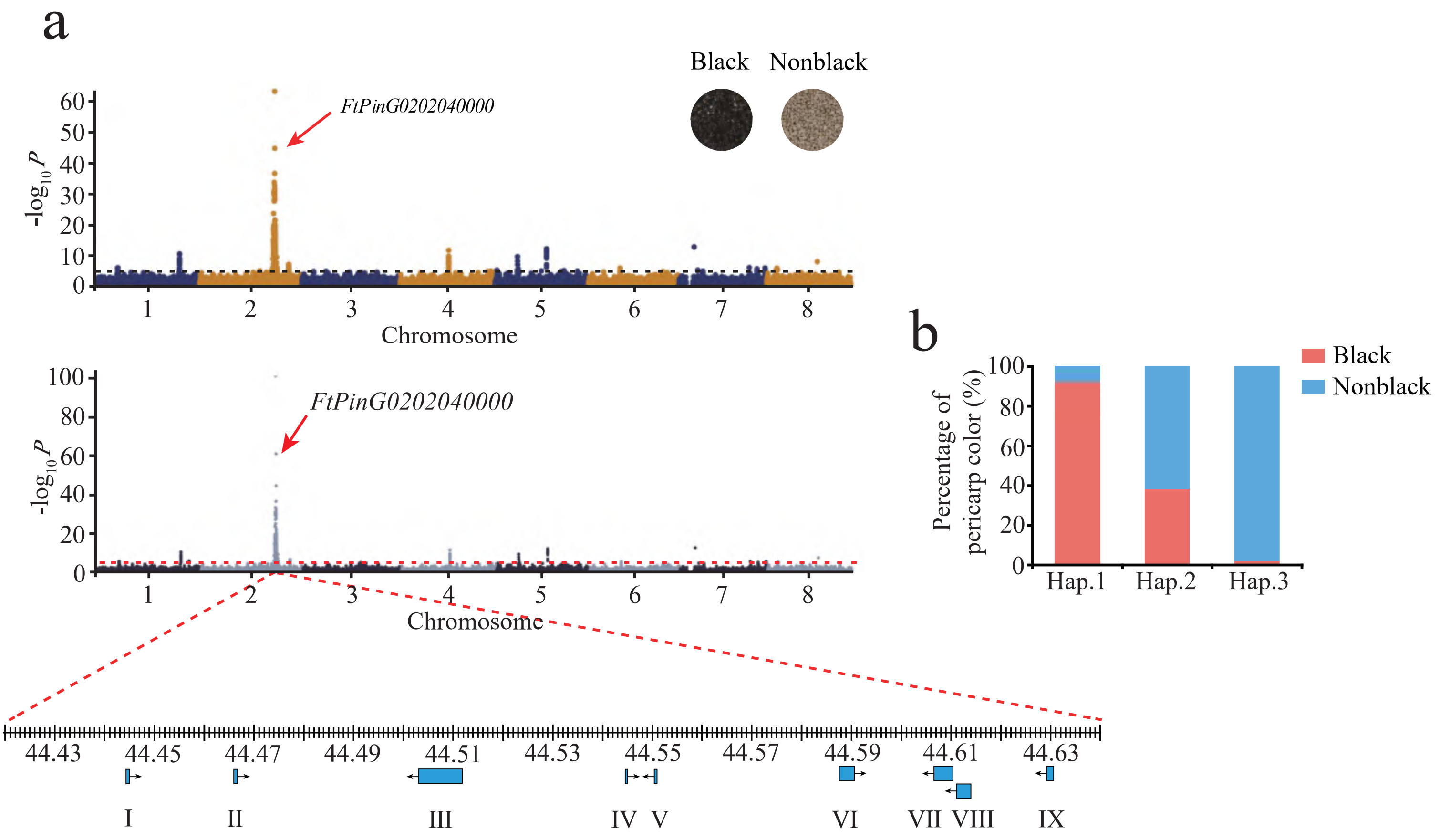

c

	Gene ID	Function in buckwheat	Homologous in Arabidopsis
I	*FtPinG0202039300*	B-box-type zinc finger	*AT1G31040* (*ORE15*)
II	*FtPinG0202039700*	Ovate protein family	*AT1G06920* (*OFP4*)
III	*FtPinG0202040000*	SANT/Myb domain	*AT5G35550* (*TT2*)
IV	*FtPinG0202040900*	Late embryogenesis abundant protein	*AT3G52470* (*LEA*)
V	*FtPinG0202041200*	Late embryogenesis abundant protein	*AT3G52470* (*LEA*)
VI	*FtPinG0202041500*	Cytokinin riboside 5'-monophosphate phosphoribohydrolase LOG	*AT5G06300* (*LOG7*)
VII	*FtPinG0202042000*	Alkyl hydroperoxide reductase subunit C	*AT3G11630* (*2CPA*)
VIII	*FtPinG0202042400*	HVA22-related protein	*AT2G36020* (*HVA22J*)
IX	*FtPinG0202042600*	Short-chain dehydrogenase/reductase SDR	*AT3G51680* (*SDR2*)

Figure S28 GWAS analysis of pericarp color. a, Manhattan plots for GWAS on pericarp color. The upper and middle manhattan plots are calculated by EMMAx and FaST-LMM, respectively. The red arrow indicates the candidate gene. The dashed line indicates the threshold -log*P* = 5. The basal image shows a 100kb region on each side of the peak SNP. b, Box plots showing PC in three haplotypes (Hap.). c, List of candidate genes in the association region (43.53-45.53 Mb). Hap., haplotype.

Figure S29

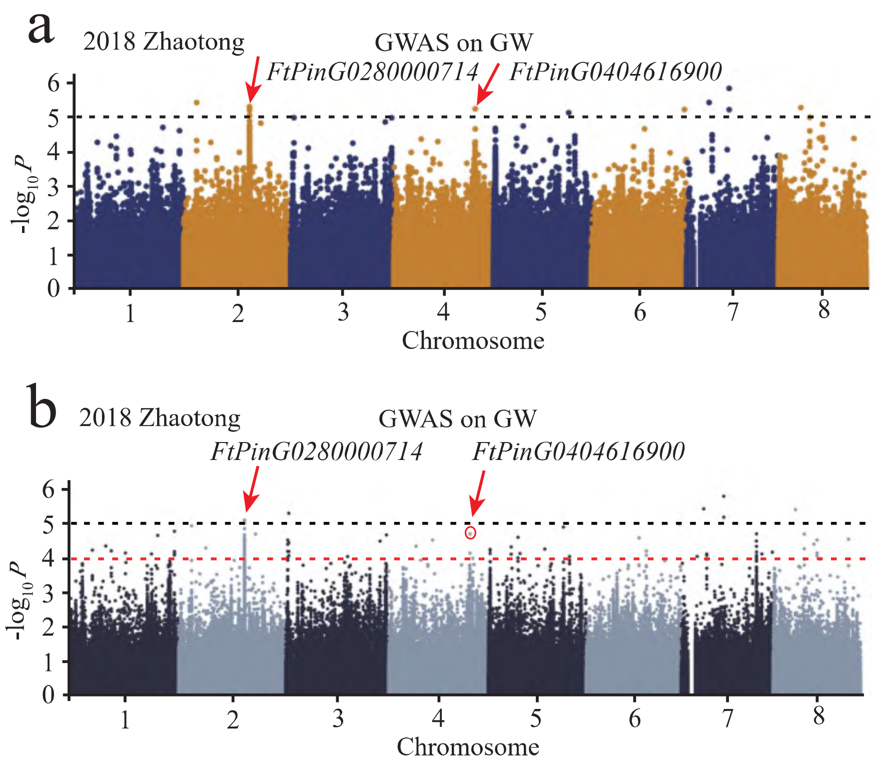
a
2018 Zhaotong
GWAS on GW
FtPinG0280000714
FtPinG0404616900
-log10 P
0
1
2
3
4
5
6
1
2
3
4
5
6
7
8
Chromosome
b
2018 Zhaotong
GWAS on GW
FtPinG0280000714
FtPinG0404616900
-log10 P
0
1
2
3
4
5
6
1
2
3
4
5
6
7
8
Chromosome

Figure S29 GWAS analysis of 1000-grain-weight. a-c, Manhattan plots for GWAS are calculated by EMMAx. d-f, Manhattan plots are calculated by FaST-LMM. Red arrows indicate the candidate genes. The black dashed line indicates the threshold -log*P* = 5. The red dashed line indicates the threshold -log*P* = 4.

Figure S30

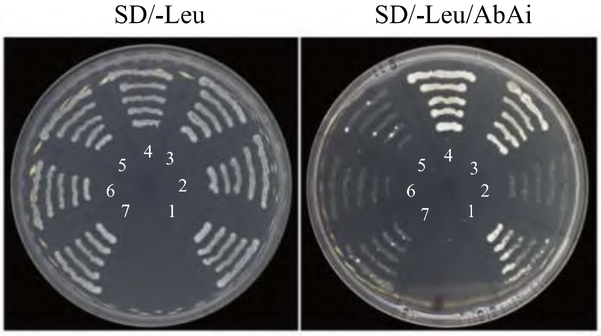

Figure S30 Y1H assay for the activity of $FtAP2YT1^{Pro}$ and $FtAP2YT1^{Ala}$ binding to GCC-box and mGCC-box. Yeast transformants were grown on the selective medium (SD-Leu/AbA). AbA, Aureobasidin A; 1-3 represent co-transformation of GCC-box-pABAi and $FtAP2YT1^{Pro}$-pGADT7, pGADT7, and $FtAP2YT1^{Ala}$-pGADT7, respectively; 4 represents co-transformation of pGBKT7-53 and pGADT7-Rec T, used as positive control; 5-7 represent co-transformation of mGCC-box-pABAi with $FtAP2YT1^{Pro}$-pGADT7, pGADT7, and $FtAP2YT1^{Ala}$-pGADT7, respectively.

Figure S31

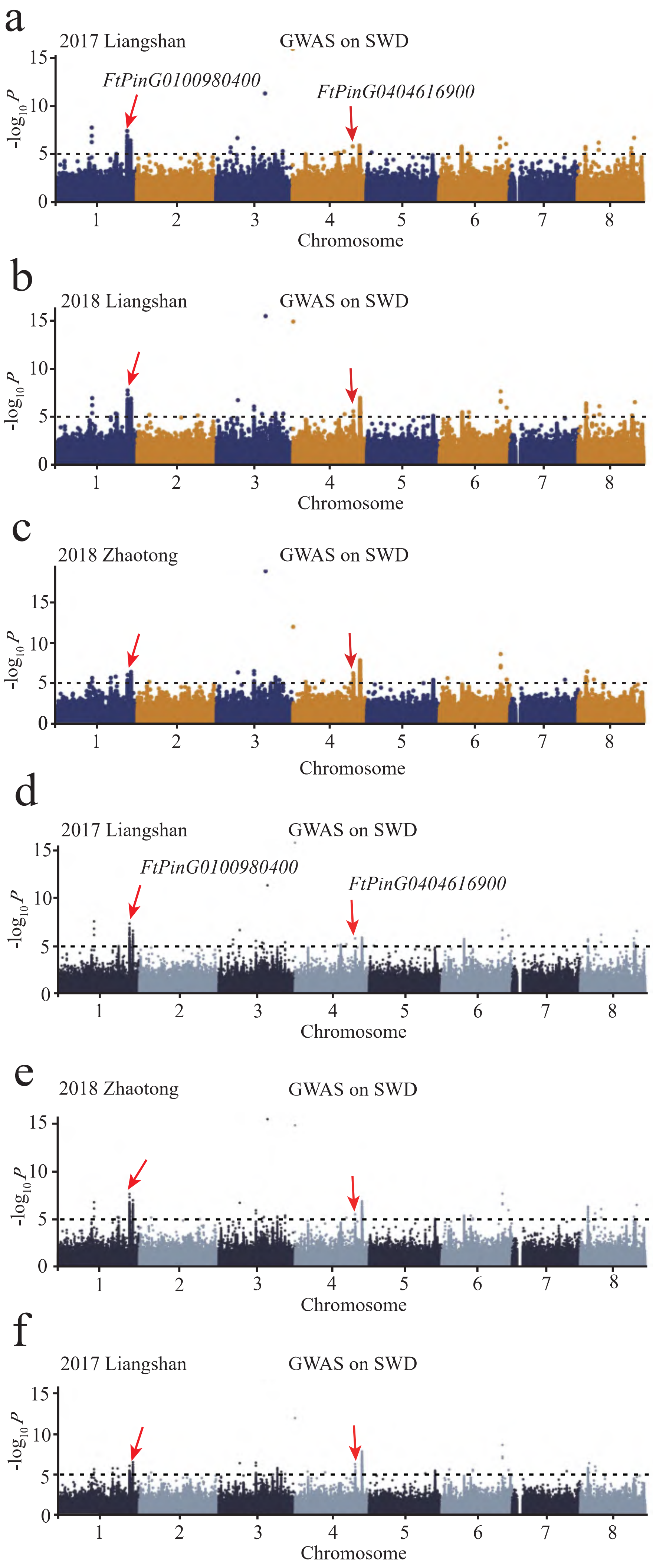

a
2017 Liangshan
GWAS on SWD
FtPinG0100980400
FtPinG0404616900
-log10P
Chromosome
b
2018 Liangshan
GWAS on SWD
c
2018 Zhaotong
GWAS on SWD
d
2017 Liangshan
GWAS on SWD
FtPinG0100980400
FtPinG0404616900
e
2018 Zhaotong
GWAS on SWD
f
2017 Liangshan
GWAS on SWD

Figure S31 GWAS analysis of seed width. a-c, Manhattan plots for GWAS are calculated by EMMAx. d-f, Manhattan plots are calculated by FaST-LMM. Red arrows indicate the candidate genes. The dashed line indicates the threshold -log$P$ = 5.

Figure S32

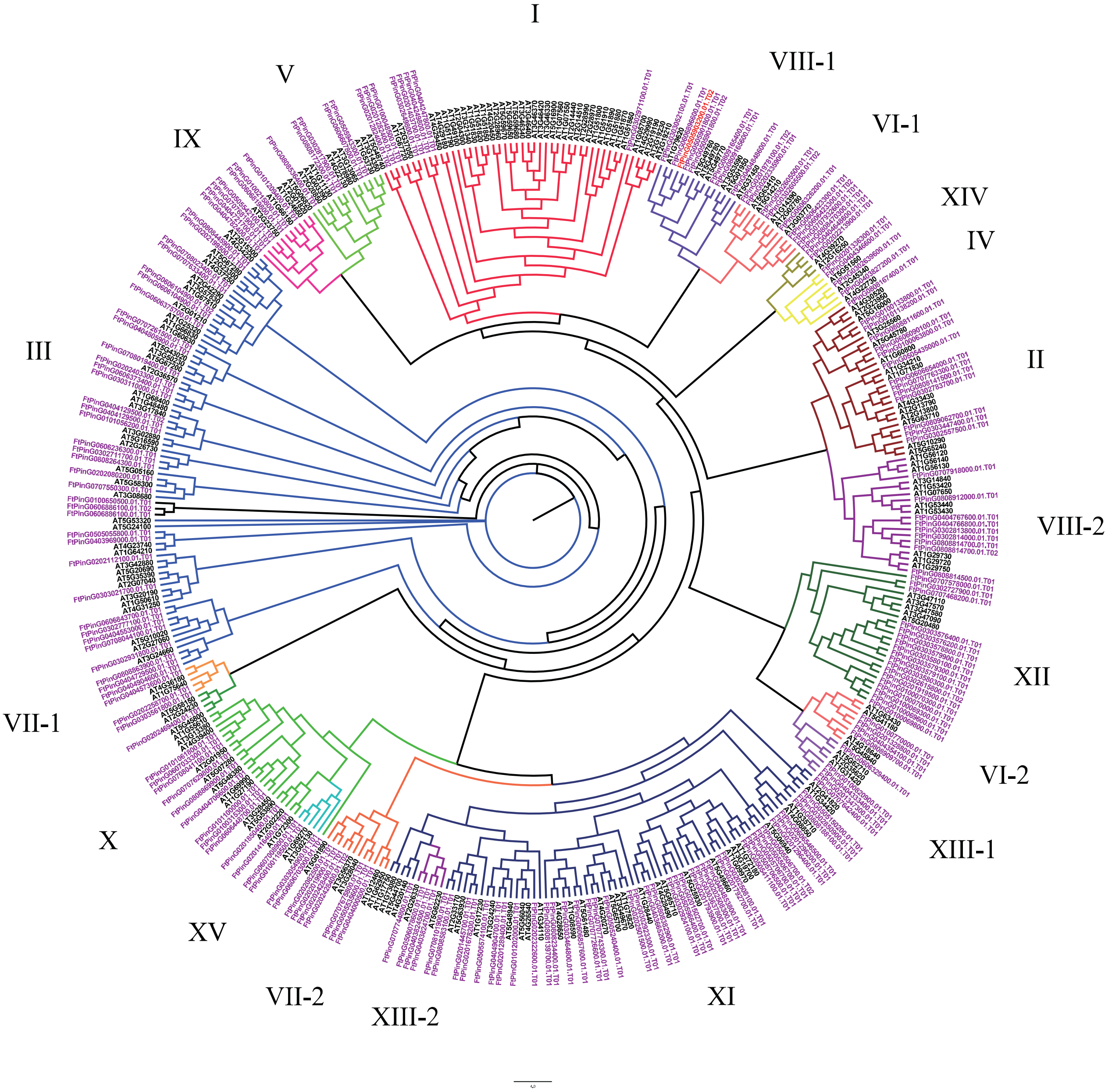
I
VIII-1
V
VI-1
IX
XIV
IV
III
II
VIII-2
XII
VII-1
VI-2
X
XIII-1
XV
XI
VII-2
XIII-2

Figure S32 Maximum likelihood tree of *LRR-RLK* genes. The maximum likelihood tree was constructed using amino acid sequences from Tartary buckwheat and *Arabidopsis thaliana*, marked in purple and black front, respectively. 199 buckwheat *LRR-RLK* genes are identified, which are classified into 19 clades with the Arabidopsis *LRR-RLKs*.
