# Supplementary material for: Resequencing of global Tartary buckwheat accessions reveals multiple domestication events and key loci associated with agronomic traits
Source: Genome Biol. 2021 Jan 12;22:23. doi: 10.1186/s13059-020-02217-7 (PMC7802136; doi:10.1186/s13059-020-02217-7)
Supplement: Supplementary file 3 — Additional file 3. Review history. [file 13059_2020_2217_MOESM3_ESM.docx]

Review History

**First round of review**

**Reviewer 1**

**Are you able to assess all statistics in the manuscript, including the appropriateness of statistical tests used?** Yes, and I have assessed the statistics in my report.

**Comments to author:**

The manuscript of Zhang et al. describes the analysis of resequencing data of a diversity panel of Tartary buckwheat accessions. The authors analyze population structure, domestication history and map traits by means of GWAS. Many of the conclusions are insufficiently supported by the data, which is in part due to the shortness of the manuscript, and part to due lack of technical validation. Much effort need to be spend in improving the manuscript and making the analyses more robust. My specific comment are:

1. The main focus of the manuscript is on GWAS. A single GWAS methods (EMMAx) was used and p-values were taken at face value. In my opinion, the signals shown in Figs. 2c-f are more likely to be spurious rather than not. A strong, and likely genuine signal for a trait with a presumably simply inheritance model is shown in Fig S22a (significance scores >60). The signals in Figs. 2c-f are barely above the significance threshold (5-10). Alternative explanations such as population structure or simply noise need to be explored. The authors should run multiple GWAS methods to assess the robustness of their signals.

2. The same concerns apply to Fig. 4a.

3. Along the same lines, multiple complementary methods in addition to XP-CLR should be applied to detect selective sweeps and assess their robustness.

4. More details are required for the overexpression assays. I am not an expert in functional genomics, but I know that transgenic approaches are extremely challenging to implement in many species. The short description in ll. 433-435 is likely insufficient to reproduce the results. The cited reference (#49, Zhou et al. 2010) describes a method in Madagascar Periwinkle, a subtropical ornamental plant with unclear relationship to Fagopyron.

5. Overexpression was performed in roots, but mass spec was done for seeds. The relevance of transcript abundance in roots for seed composition need to be explained.

6. The manuscript is too short in general. The introduction does not establish the current state-of-the art in Fagopyron genomics. The research is not put into wider context.

7. The conclusion in ll. 212-214 is very weak. Functional investigation (which is hard) is not required for marker development, which can be done by genetic mapping with medium-density marker platforms such as GBS or arrays.

8. The traits "seed wing" show a very promising GWAS signal. But this is not followed up. The meaning of "seed wing" is not explained.

9. L. 190: LRR homologs are very abundant in all plant genomes. A more detailed analysis than simple homology searches are required to establish a meaningful relationship.

**Reviewer 2**

**Are you able to assess all statistics in the manuscript, including the appropriateness of statistical tests used?** There are no statistics in the manuscript.

**Comments to author:**

Zhang et al. resequenced 510 accessions of Tartary buckwheat, including 478 landraces, 32 wild accessions, and seven other Fagopyrum genus species. The study aimed to explore the population structure, domestication events, and also performed the genome-wide association studies for critical agricultural traits. They identified several candidate genes significantly correlated to flavonoid accumulation and grain weight. The topics addressed are interesting, but there are several problems with the manuscript as presented. Some of the conclusions are lack of evidence to support. There are several issues with the manuscript that need to be addressed before the manuscript is published.

Major issues:

1. The authors concluded that a likely independent domestication process is driven by human intervention in the two genetically and geographically distinct groups (SL and NL). The conclusion is only based on very few overlapped selective sweep regions or genes identified by XP-CLR between SL and NL groups. This is not convincible, because Mat et al., 2015 suggested that XP-CLR produces signals in the selected position, but almost equally strong signals in other positions as well (https://doi.org/10.1038/hdy.2015.42). If the author just based on XP-CLR, may add more false positive regions and get wrong conclusion. I would use de-correlated composite of multiple signals (DCMS) approach as Mat et al., 2015 developed to see how many overlapped selective regions between those two groups.

2. Again, the authors also calculated the population differentiation FST between SL and NL. How many regions identified by FST approaches overlapped with the SL or NL unique selective sweep regions?

3. The GWAS results seem disconnected from the main story of the domestication. How do those genes identify to associated with the flavonoid metabolism or agronomic traits related to the selective sweep regions? Are all of them located in the selective sweep regions?

4. About SNP calling and validation, in line 368-370, the author removed the SNPs shown as the three clustered in a ten bp windows. Please explain the rationale. The authors only sample 32 out of the 1 million SNPs to do validation, that is too tinny. Could the author tested more, for example, 0.1% of the SNPs (1,000), to do validation? To see if the SNPs called from whole-genome resequencing data are reasonable, please show the site frequency spectrum (SFS) (both folded and unfolded).

5. The authors used VCFtools to calculate Fst.

6. VCFtools were designed to calculate the population genetic statistics for diploid samples. Still, the majority of the samples in this study are highly selfing (which will be treated as haploid), and can not VCFtools to calculate Fst.

7. All of the scripts and analytical tools used for a largely computational project like the work reported here need to be made available through Github, Bitbucket, or a similar version control repository. Because the scripts are essential to reproducing this work, they should be available to reviewers and/or readers. These resources are important in part because samtools, GATK, and similar utilities include a large number of options that alter the resulting output.

8. Some of the arguments or conclusions seem to be lack of interpretation/clarification. For example, in Line 121-123, the authors argued that "This 1.77 ratio…" Could the author make some interpretations of this pattern? Also, how about the Ts/Tv ratios compared with other major crops? Line 148-152, Where is the center of origin correctly based on the authors' data? Both southwestern China and Himalaya or just Himalaya? Line 154-157, "only three accessions, were all grouped with wild accessions", what do you mean wild accessions? Do you expect the HW group? It isn't very clear because each of the three groups (HW, NL, and SL)

9. About wild accessions. Also, are the wild accessions the same subspecies with the domesticated species? Why does each group include wild accessions? What is the population structure of the wild accessions like? Also, three groups?

10. Did the author check if the orthologues of "famous" genes related to domestication, for example, shattering genes or genes related to the domesticated syndrome (Meyer et al., 2012, https://doi.org/10.1111/j.1469-8137.2012.04253.x) located in the selective sweep regions identified by this study?

Minor issues:

Line202, what do you mean of "differentiation sweep"? If any literature defined, please cite.

**Reviewer 3**

**Are you able to assess all statistics in the manuscript, including the appropriateness of statistical tests used?** Yes, and I have assessed the statistics in my report.

**Comments to author:**

This study by Zhang et al. conducted the first(?) population genomic analysis of Tartary buckwheat. They whole genome sequenced over 500 lines including landraces and wild varities.They then conduct a population genomic analysis to find regions of selective sweep using XP-CLR which is looking at population differences in allele frequency. They use this result to interpret the domestication history of Tartary buckwheat. They also conducted a GWA to find out the genetic architecture underlying important agronomic traits of Tartary buckwheat. Overall I think this is a pretty good analysis and given the understudied status of Tartary buckwheat I think this is a great study that will open the doors for future Tartary ag research. The main concern I have is with their population genetic analysis and by addressing my concerns I think this is a great study for Genome Bio.

Major comment

1. The authors have indicated in multiple places of the manuscript that they determined the origin of Tartary buckwheat domestication (L148, L307). Even mentioning it as a main result by writing it in the abstract (L72). However I dont see any results that support this claim. What analysis specifically addressed this question? At least in the rice and maize domestication field, they do simulations and demographic modeling to specifically test the geographic origin or the population origin of a domesticate. Or studies compare hypothesized wild progenitor to domsticate to infer origins. The high quality of the data generated by the authors are no different from the rice or maize people. But here I dont see any analysis that specifically addressed the origin question. I think the only analysis I see is the phylogenetic relationship and geographic distribution. None of this is actually testing origins and I suggest the authors either roll back on their comments on this matter in their manuscript, unless they will address this question by specifically testing the origins of domestication for Tartary buckwheat.

2. The authors claim evidence of independent domestication for NL vs. SL but this again falls short of the analysis required to suggest this case. Having less windows of overlapping XP-CLR is not sufficient to say that NL and SL have independent origins. In fact all the XP-CLR peaks could have purely driven by demographic histories given the bottlenecking involved with domestication reduces the levels of polymorphism. Again there needs to be more direct evidence or specific tests to show that indeed there has been absolute independent origins of NL vs. SL. One thing that came to my mind was to combine the GWAS and selective sweep results together. Sweeps are population genetic evidence of a domestication event while GWAS is the quantitative genetic evidence of a domestication event. In otherwords looking for genomic regions that are associated with important Tartary buckwheat agronomic traits and see if they also have the population genetic evidence of a selective sweep. This is then a good evidence that said region was involved in buckwheat domestication and not purely due to neutral demographic events completely unrelated to the domestication genetics. Then one would have to look at the phylogenetic relationship of said candidate domestication regions to test if potential causal domestication mutation/haplotype has a single origin or independent origin.

I would highly suggest the authors to read the study by Huang et al. 2012 Nature (https://www.nature.com/articles/nature11532), as this study has similar data types to Huang etal. but Huang et al. has specifically tested the domestication models of rice to infer whether the crop was independently domesticated and even where the crop was geographically domesticated etc. I think the authors have the data but I would argue they haven't shown any convincing results showing the domestication model of Tartary buckwheat.

3. This is a minor/major comment but I think in subsequent revision steps I would like to see the authors upload the codes, scripts, and data that was generated from this study. Nowadays there's really no excuse to not show the computational work that was done for a project. For instance using github to show the commands that were taken for the analysis of this paper, and the raw data such as VCFs or other intermediate files generated from the analysis (not saying to upload all but just the key files that will be useful for people who read this paper and want to use their dataset). For raw data there's dryad (nominal fee) or zenodo (completely free) and I highly urge the authors to upload key datasets generated from this study onto these repositories because once its uploaded it will never disappear.

**Authors Response**

**Point-by-point responses to the reviewers’ comments:**

**Reviewer #1**:

Comment 1. The main focus of the manuscript is on GWAS. A single GWAS

methods (EMMAx) was used and p-values were taken at face value. In my opinion, the

signals shown in Figs. 2c-f are more likely to be spurious rather than not. A strong, and

likely genuine signal for a trait with a presumably simply inheritance model is shown

in Fig S22a (significance scores >60). The signals in Figs. 2c-f are barely above the

significance threshold (5-10). Alternative explanations such as population structure or

simply noise need to be explored. The authors should run multiple GWAS methods to

assess the robustness of their signals.

*Response 1: We applied the FastLMM model together with the EMMAX, all the*

*GWAS signals addressed in the manuscript were generated by both models (Additional*

*Personal Cover Click here to access/download;Personal Cover;cover letter for*

*revision_2020.6.2.docx*

*file 1: Figure S13, S20-S22, S26 and S28), although there are many expected different*

*signals from the two methods. The single GWAS method has been successfully used in*

*a lot of publications since 2015, such as soybean (Zhou et al. 2015 Nature*

*Biotechnology; Fang et al. 2017 Genome Biology), rice (Si et al. 2016 Nature Genetics),*

*cotton (Du et al. 2018 Nature Genetics), watermelon (Guo et al. 2019 Nature Genetics),*

*melon (Zhao et al. 2019 Nature Genetics), date palm (Hazzouri et al. 2019 Nature*

*Communication), wheat (Chen et al. 2020 Plant Biotechnology Journal). Therefore, we*

*think results from either EMMAX or FastLMM are reliable in our research.*

Comment 2. The same concerns apply to Fig. 4a.

*Response 2: Both EMMAX and FastLMM models identified this signal (Additional*

*file 1: Figure S26).*

Comment 3. Along the same lines, multiple complementary methods in addition to

XP-CLR should be applied to detect selective sweeps and assess their robustness.

*Response 3: We performed the de-correlated composite of multiple signals (DCMS)*

*approach in addition to XP-CLR (Additional file 1: Figure S14), on top of 10%, we*

*identified 146 sweeps and 112 sweeps in SL and NL, and 59% and 68% overlapping*

*sweeps compared with XP-CLR, respectively, were identified between the two methods*

*(Additional file 2: Table S7, S8, S10 and S11). Moreover, 22 GWAS signals overlapped*

*in both methods (9 in SL and 13 in NL) for 7 traits (Additional file 2: Table S12).*

Comment 4. More details are required for the overexpression assays. I am not an

expert in functional genomics, but I know that transgenic approaches are extremely

challenging to implement in many species. The short description in ll. 433-435 is likely

insufficient to reproduce the results. The cited reference (#49, Zhou et al. 2010)

describes a method in Madagascar Periwinkle, a subtropical ornamental plant with

unclear relationship to Fagopyron.

*Response 4: Buckwheat is the one that has much challenges in transgenic*

*approaches and we have been trying our best to do it. Our previous publications in*

*New Phytologist (Zhou et al. 2017), Journal of Experimental Botany (Zhang et al. 2018)*

*and Plant Biotechnology Journal (Li et al. 2019) have shown that buckwheat hairy root*

*system is a fast and effective approach for the gene function analysis, especially for the*

*genes involved in secondary metabolisms. We cited the three references for this part*

*and described a detailed procedure for the generation of transgenic hairy roots.*

Comment 5. Overexpression was performed in roots, but mass spec was done for

seeds. The relevance of transcript abundance in roots for seed composition need to be

explained.

*Response 5: The flavonoid accumulation occurs in almost all buckwheat tissues. The*

*overexpression in hairy root is only for the gene functional analysis. The transcript*

*levels were tested in different stages of seed development and in different haplotypes,*

*not in the roots.*

Comment 6. The manuscript is too short in general. The introduction does not

establish the current state-of-the art in Fagopyron genomics. The research is not put

into wider context.

*Response 6: We reorganized the structure of the manuscript. Results from new*

*analysis were added in the results, discussion and introduction were rewritten and the*

*state-of-the-art in Fagopyrum genomics were added in the introduction part.*

Comment 7. The conclusion in ll. 212-214 is very weak. Functional investigation

(which is hard) is not required for marker development, which can be done by genetic

mapping with medium-density marker platforms such as GBS or arrays.

*Response 7: This part was revised following the comment.*

Comment 8. The traits "seed wing" show a very promising GWAS signal. But this is

not followed up. The meaning of "seed wing" is not explained.

*Response 8: Seed wing is a special character of buckwheat pericarp (shown in the*

*picture), which might be used for its dispersal by wind and prevent being eaten by birds.*

*The mechanism of its formation is unknown and difficult to be uncovered, due to the*

*unavailability of Tartary buckwheat mutant. In this article, we mainly focus on the*

*quality and yield traits for the Tartary buckwheat improvement, so we didn’t follow up*

*this promising GWAS signal. In the future study, we will look through this signal and*

*perform further analysis.*

Comment 9. L. 190: LRR homologs are very abundant in all plant genomes. A more

detailed analysis than simple homology searches are required to establish a meaningful

relationship.

*Response 9: We aligned 199 Tartary buckwheat LRR-RLKs with Arabidopsis, which*

*were divided into 19 subfamilies. The gene FtPinG0505903200 associated with plant*

*height in the selective sweep of SL was clustered in the VIII-1 group (Additional file 1:*

*Figure S32). Detailed functional analysis will be performed in the future studies.*

**Reviewer #2:**

Comment 1. The authors concluded that a likely independent domestication process

is driven by human intervention in the two genetically and geographically distinct

groups (SL and NL). The conclusion is only based on very few overlapped selective

sweep regions or genes identified by XP-CLR between SL and NL groups. This is not

convincible, because Mat et al., 2015 suggested that XP-CLR produces signals in the

selected position, but almost equally strong signals in other positions as well

(https://doi.org/10.1038/hdy.2015.42). If the author just based on XP-CLR, may add

more false positive regions and get wrong conclusion. I would use de-correlated

composite of multiple signals (DCMS) approach as Mat et al., 2015 developed to see

how many overlapped selective regions between those two groups.

*Response 1: De-correlated composite of multiple signals (DCMS) approach was*

*performed in addition to XP-CLR (Additional file 1: Figure S14), on top of 10%, we*

*identified 146 sweeps in SL and 112 sweeps NL. As expected, different methods*

*generated different sweeps, however 59% and 68% overlapping sweeps were found in*

*SL and NL, respectively. Moreover, 22 GWAS signals overlapped in both methods (9*

*in SL and 13 in NL) for 7 traits (Additional file 2: Table S12).*

Comment 2. Again, the authors also calculated the population differentiation FST

between SL and NL. How many regions identified by FST approaches overlapped with

the SL or NL unique selective sweep regions?

*Response 2: The comparison of genetic diversity (πwild/πlandrace) combined with FST*

*were used to look for the unique selective sweep regions in SL and NL (Additional file*

*1: Figure S18). We identified 4 unique selective sweeps in SL and 8 in NL (Additional*

*file 2: Table S17).*

Comment 3. The GWAS results seem disconnected from the main story of the

domestication. How do those genes identify to associated with the flavonoid

metabolism or agronomic traits related to the selective sweep regions? Are all of them

located in the selective sweep regions?

*Response 3: Now, for the buckwheat breeding and production, quality and yield are*

*the most important traits. The 1000-grain weight partially reflecting yield increased in*

*landraces (Additional file 1: Figure S6), and we do find some genes associated with*

*1000-grain weight and plant height in the selective sweep regions (Fig. 2), remaining*

*to be further analyzed. Although we didn’t find the connection between the current*

*GWAS genes in Fig.3 for flavonoids and Fig.4 for yield and domestication, their*

*promising functions caught our attention. Especially, the nonsynonymous SNP in the*

*exonic region of the FtAP2YT1 gene led to the change of protein structure and affected*

*its DNA binding function, which is a very interesting discovery.*

Comment 4. About SNP calling and validation, in line 368-370, the author removed

the SNPs shown as the three clustered in a ten bp windows. Please explain the rationale.

The authors only sample 32 out of the 1 million SNPs to do validation, that is too tinny.

Could the author tested more, for example, 0.1% of the SNPs (1,000), to do validation?

To see if the SNPs called from whole-genome resequencing data are reasonable, please

show the site frequency spectrum (SFS) (both folded and unfolded).

*Response 4: In total, 510 SNPs were validated and the accuracy was estimated to be*

*95.1% (Additional file 2: Table S4). The site frequency spectrum (SFS) were added in*

*the result (Additional file 1: Figure S3).*

Comment 5. The authors used VCFtools to calculate Fst.

Comment 6. VCFtools were designed to calculate the population genetic statistics

for diploid samples. Still, the majority of the samples in this study are highly selfing

(which will be treated as haploid), and can not VCFtools to calculate Fst.

*Response 5&6: PopGenome was used for the calculation and new results were*

*shown in Additional file 1: Figure S18.*

Comment 7. All of the scripts and analytical tools used for a largely computational

project like the work reported here need to be made available through Github, Bitbucket,

or a similar version control repository. Because the scripts are essential to reproducing

this work, they should be available to reviewers and/or readers. These resources are

important in part because samtools, GATK, and similar utilities include a large number

of options that alter the resulting output.

*Response 7: We have uploaded of the codes, scripts, and sequencing data generated*

*from this study in Github (https://github.com/rahello/tartary_population) and NCBI*

*(PRJNA600676).*

Comment 8. Some of the arguments or conclusions seem to be lack of

interpretation/clarification. For example, in Line 121-123, the authors argued that "This

1.77 ratio…" Could the author make some interpretations of this pattern? Also, how

about the Ts/Tv ratios compared with other major crops? Line 148-152, Where is the

center of origin correctly based on the authors' data? Both southwestern China and

Himalaya or just Himalaya? Line 154-157, "only three accessions, were all grouped

with wild accessions", what do you mean wild accessions? Do you expect the HW

group? It isn't very clear because each of the three groups (HW, NL, and SL)

*Response 8: More detailed introduction/clarification/interpretation were added in*

*the manuscript to support the arguments or conclusions. Ts/Tv ratios were added in the*

*results. The wild accessions in HW group include samples from Tibet, India, Bhutan*

*and Nepal, although wild samples from Sichuan, Yunnan and Guizhou were not include*

*in this research owing to enormous difficulties in collecting wild Tartary buckwheat*

*that normally growing in high attitude mountain areas. Tibet locates in the*

*southwestern China where belongs to the Himalayan region, so our results confirmed*

*that Himalayan region is the origin place.*

Comment 9. About wild accessions. Also, are the wild accessions the same

subspecies with the domesticated species? Why does each group include wild

accessions? What is the population structure of the wild accessions like? Also, three

groups?

*Response 9: Wild accessions (HW group) here are Tartary buckwheat collected from*

*the wild, but not the wild ancestors or wild relatives of Tartary buckwheat. Wild*

*accessions in SL and NL result from the incorrect definition of the accession name in*

*the Genebank, which were modified in this revised manuscript. We cannot clearly see*

*the population structure of wild accessions because of the small number of accessions.*

*For the next step, the collection of wild accessions and wild relatives of Tartary*

*buckwheat will be one of the most important part of our studies.*

Comment 10. Did the author check if the orthologues of "famous" genes related to

domestication, for example, shattering genes or genes related to the domesticated

syndrome (Meyer et al., 2012, https://doi.org/10.1111/j.1469-8137.2012.04253.x)

located in the selective sweep regions identified by this study?

*Response 10: Unlike the main crops, rice, wheat and maize, buckwheat still exhibits*

*server seed shattering, thick shell, tall stalk, plant lodging and low yield, indicating*

*that it is still on the primary stage of domestication. Therefore, those famous genes*

*associated with typical domesticated traits, such as sh4 for seed shattering of rice, tga1*

*for naked grains of maize, PROG1 for tiller angle of rice, G for seed dormancy in*

*soybean, rice and tomato, were not found in the genetically selective sweeps obtained*

*by multiple methods.*

Minor issue: Line202, what do you mean of "differentiation sweep"? If any

literature defined, please cite.

*Response 11: It was revised following the comment.*

**Reviewer #3**:

Comment 1 The authors have indicated in multiple places of the manuscript that they

determined the origin of Tartary buckwheat domestication (L148, L307). Even

mentioning it as a main result by writing it in the abstract (L72). However, I dont see

any results that support this claim. What analysis specifically addressed this question?

At least in the rice and maize domestication field, they do simulations and demographic

modeling to specifically test the geographic origin or the population origin of a

domesticate. Or studies compare hypothesized wild progenitor to domsticate to infer

origins. The high quality of the data generated by the authors are no different from the

rice or maize people. But here I dont see any analysis that specifically addressed the

origin question. I think the only analysis I see is the phylogenetic relationship and

geographic distribution. None of this is actually testing origins and I suggest the authors

either roll back on their comments on this matter in their manuscript, unless they will

address this question by specifically testing the origins of domestication for Tartary

buckwheat.

*Response 1: We rolled back our results, reorganized our conclusions and added*

*more introduction and references in the revised manuscript. The origin of buckwheat*

*has been widely accepted in Himalayan region, owing to the rich genetic diversity of*

*Fagopyrum resources and the characterization of cultivated buckwheat ancestors. The*

*phylogenetic relationship in our results partially confirmed that Himalayan region is*

*the origin place. In order to reveal the origin, a large number of wild accessions and*

*wild relatives of Tartary buckwheat are required, the dynamic simulation and*

*distribution of buckwheat population may be provided as supplementary information.*

Comment 2. The authors claim evidence of independent domestication for NL vs. SL

but this again falls short of the analysis required to suggest this case. Having less

windows of overlapping XP-CLR is not sufficient to say that NL and SL have

independent origins. In fact all the XP-CLR peaks could have purely driven by

demographic histories given the bottlenecking involved with domestication reduces the

levels of polymorphism. Again there needs to be more direct evidence or specific tests

to show that indeed there has been absolute independent origins of NL vs. SL. One

thing that came to my mind was to combine the GWAS and selective sweep results

together. Sweeps are population genetic evidence of a domestication event while

GWAS is the quantitative genetic evidence of a domestication event. In otherwords

looking for genomic regions that are associated with important Tartary buckwheat

agronomic traits and see if they also have the population genetic evidence of a selective

sweep. This is then a good evidence that said region was involved in buckwheat

domestication and not purely due to neutral demographic events completely unrelated

to the domestication genetics. Then one would have to look at the phylogenetic

relationship of said candidate domestication regions to test if potential causal

domestication mutation/haplotype has a single origin or independent origin. I would

highly suggest the authors to read the study by Huang et al. 2012 Nature

(https://www.nature.com/articles/nature11532), as this study has similar data types to

Huang etal. but Huang et al. has specifically tested the domestication models of rice to

infer whether the crop was independently domesticated and even where the crop was

geographically domesticated etc. I think the authors have the data but I would argue

they haven't shown any convincing results showing the domestication model of Tartary

buckwheat.

*Response 2: We performed the de-correlated composite of multiple signals (DCMS)*

*approach in addition to XP-CLR following the advice. As expected, different methods*

*generated different sweeps, however 59% and 68% overlapping sweeps were found in*

*SL and NL, respectively. Moreover, GWAS and selective sweep results were combined,*

*and 22 GWAS signals overlapped in both methods (9 in SL and 13 in NL) for 7 traits*

*(Additional file 2: Table S12). The comparison of genetic diversity (πwild/πlandrace)*

*combined with FST were used to look for the unique selective sweep regions in SL and*

*NL (Additional file 1: Figure S18). We identified 4 unique selective sweeps in SL and 8*

*in NL (Additional file 2: Table S17). Genetic variations in the selective sweeps were*

*used for the phylogenetic relationship of candidate domestication regions, we can see*

*clearly two populations (SL and NL, shown below) similar to the structure generated*

*from the whole-genome.*

*Buckwheat still exhibits server seed shattering, thick shell, tall stalk, plant lodging*

*and low yield, and those famous genes associated with typical domesticated traits were*

*not found in the selective sweep regions, indicating that it is still on the primary stage*

*of domestication compared to the main crops, rice, wheat and maize. Because of the*

*small number of wild accessions, in this research the population structure of HW*

*cannot be divided to different subgroups like rice study by Huang et al. 2012 Nature.*

*Wild accessions (HW group) here are collected from the wild, but not the wild ancestors*

*or wild relatives of Tartary buckwheat. During the natural and human selection in*

*terms of different environmental conditions and preference, the superimposition of*

*selective pressure created the different selective sweeps and loci in different groups.*

Comment 3. This is a minor/major comment but I think in subsequent revision steps

I would like to see the authors upload the codes, scripts, and data that was generated

from this study. Nowadays there's really no excuse to not show the computational work

that was done for a project. For instance using github to show the commands that were

taken for the analysis of this paper, and the raw data such as VCFs or other intermediate

files generated from the analysis (not saying to upload all but just the key files that will

be useful for people who read this paper and want to use their dataset). For raw data

there's dryad (nominal fee) or zenodo (completely free) and I highly urge the authors to

upload key datasets generated from this study onto these repositories because once its

uploaded it will never disappear.

*Response 3. We have uploaded of the codes, scripts, and sequencing data generated*

*from this study in Github (https://github.com/rahello/tartary_population) and NCBI*

*(PRJNA600676).*

**Second round of review**

**Reviewer 1**

The authors have prepared a detailed response and removed my concerns about the technical correctness of their GWAS analyses.

But I am not convinced that the concern of the other reviewers about domestication-related claim are properly resolved. I believe more careful demographic analyses are needed to corroborate the claim of independent domestication events.

The best solution may be to remove claims about domestication history. Conducting a GWAS study in a minor crop and finding strong peaks is still a major achievement.

**Reviewer 2**

Zhang et al. solved majority of the issues I've addressed, but there are still several issues not clarified.

Major:

About Figure S3, the authors plotted both SFS and unfolded SFS. The pattern of both looks very strange. Why the rare alleles have such low frequency? Presumably, the rare alleles always have the highest frequency. The patterns are supposed to be like Figure 4 in Nielsen et al., 2012 (doi:10.1371/journal.pone.0037558). I suspect the authors still keep a considerable amount of false-positive SNPs. So I would set a more stringent threshold to do filtering and redo all of the downstream analysis.

The section of the "Independent domestication and divergence between SL and NL" seems disorganized. I would incorporate some of the second paragraph on page 11 into the XPCLR one. Moreover, the overlaps between the GWAS results and the SSW regions can be listed as a separate paragraph behind the GWAS section. I would put all of the content related to selective sweep in the second paragraph on page 12 ahead of the GWAS part. Then after the GWAS part, discuss the overlaps between the GWAS and SSW regions detected by different approaches (including unique SSW regions by Fst and the comparison of genetic diversity.

Minor:

I still feel confused about the why the authors need to filter the SNPs more than 3SNPS clustered in a 10-bp window. Please explain the rationale.

**Reviewer 3**

Thank you for addressing my comments. I have no further comments.

**Authors Response**

**Point-by-point responses to the reviewers’ comments:**

**Reviewer #1**: The authors have prepared a detailed response and removed my concerns

about the technical correctness of their GWAS analyses.

But I am not convinced that the concern of the other reviewers about domesticationrelated claim are properly resolved. I believe more careful demographic analyses are needed to corroborate the claim of independent domestication events. The best solution may be to remove claims about domestication history. Conducting a GWAS study in a minor crop and finding strong peaks is still a major achievement.

*Response: Thank you for your comments. In this research, our results revealed some genomic loci that shape the domestication and divergence between SL and NL, which provides resources for genomic-assisted breeding of Tartary buckwheat. This section preliminary breaks the bottleneck of Tartary buckwheat domestication and was accepted by the other two reviewers and the editor. We have to admit that the small number of wild accessions creates the major limitation for the deep study of Tartary*

*buckwheat domestication. At present, the enormous difficulty in collecting wild Tartary buckwheat and its wild relatives comes from the distribution in the high-altitude places and harsh conditions. Our research group is collecting wild buckwheat resources as much as possible every year. One of the main focuses of our future work is to elucidate the domestication story of Tartary buckwheat by detailed analysis of more wild accessions. Therefore, we would keep the claims about domestication history according to the comments of the other reviewers and the editor, which will keep the structure of*

*the manuscript integrated and provide a reference for the other buckwheat researchers.*

**Reviewer #2**: Zhang et al. solved majority of the issues I've addressed, but there are still several issues not clarified.

Major:

About Figure S3, the authors plotted both SFS and unfolded SFS. The pattern of both looks very strange. Why the rare alleles have such low frequency? Presumably, the rare alleles always have the highest frequency. The patterns are supposed to be like Figure 4 in Nielsen et al., 2012 (doi:10.1371/journal.pone.0037558). I suspect the authors still keep a considerable amount of false-positive SNPs. So I would set a more stringent threshold to do filtering and redo all of the downstream analysis.

*Response: SFS in Fig.S3 were generated from SNPs of 510 accessions filtered by hard filter parameter (--filterExpression "MQ0 >= 4 && ((MQ0 / (1.0 * DP)) > 0.1)" -- filterName "HARD_TO_VALIDATE" --filterExpression "DP < 5 " –filterName "LowCoverage" --filterExpression "QD < 1.5 " --filterName "LowQD" -- filterExpression "QUAL < 30.0" --filterName "VeryLowQual"—filterExpression "QUAL > 30.0 && QUAL < 50.0 " --filterName "LowQual" --clusterWindowSize 10-- cluster-size 3), after that we filtered callset at population level based on MAF>0.05 and missing rate<0.1, so the rare alleles showed low frequency. We plot all SNPs after hard filtration (shown below), the rare alleles exhibited similar pattern with Figure 4 in Nielsen et al., 2012. The two results have no conflict, so we would like to keep the original Fig.S3.*

The section of the "Independent domestication and divergence between SL and NL" seems disorganized. I would incorporate some of the second paragraph on page 11 into the XPCLR one. Moreover, the overlaps between the GWAS results and the SSW regions can be listed as a separate paragraph behind the GWAS section. I would put all of the content related to selective sweep in the second paragraph on page 12 ahead of the GWAS part. Then after the GWAS part, discuss the overlaps between the GWAS and SSW regions detected by different approaches (including unique SSW regions by Fst and the comparison of genetic diversity.

*Response: Thanks for your constructive suggestion, we reorganized results in this section. All the content related to selective sweeps were moved to the first and second paragraph, followed with the GWAS signals obtained from the alignment with different approaches. The last part is the description of candidate genes.*

Minor:

I still feel confused about the why the authors need to filter the SNPs more than 3SNPS clustered in a 10-bp window. Please explain the rationale.

*Response: We are working on a non-model organism for which there is no SNP data available. So following GATK best practice tutorial, we chose Hard Filtering instead*

*of Variant Recalibration (VQSR) method to filter our variants callset (*[*https://gatk.broadinstitute.org/hc/en-us/articles/360036434492-VariantFiltration*](https://gatk.broadinstitute.org/hc/en-us/articles/360036434492-VariantFiltration)*). This parameter mark clusters of at least 3 SNPs that are within a window of 10 bases between them to avoid false positive sites. Please note that we selected these hard filtering values in attempting to optimize both high sensitivity and specificity together. We applied this analysis using the ‘clusterWindowSize10’ recommended by the GATK toolkit, 3 SNPs clustered in a 10-bp window is considered to be unreliable and would generate false positive effects, for more references see Bowen et al., 2011 and 2012,*

*doi:10.1371/journal.pgen.1002050; doi.org/10.1534/genetics.111.136069.*

**Reviewer #3**: Thank you for addressing my comments. I have no further comments.

Response: *Thank you for accepting our responses and revisions.*

**Third round of review**

**Reviewer 2**

Zhang et al. solved majority of the issues I've addressed, but there are still a couple of issues not clarified.

Major:

About Figure S3, the authors plotted both SFS and unfolded SFS. The pattern of both looks very strange. Why the rare alleles have such low frequency? Presumably, the rare alleles always have the highest frequency. The patterns are supposed to be like Figure 4 in Nielsen et al., 2012 (doi:10.1371/journal.pone.0037558). I suspect the authors still keep a considerable amount of false-positive SNPs. So I would set a more stringent threshold to do filtering and redo all of the downstream analysis.

Response: SFS in Fig.S3 were generated from SNPs of 510 accessions filtered by hard filter parameter (--filterExpression "MQ0 >= 4 && ((MQ0 / (1.0 * DP)) > 0.1)" -- filterName "HARD_TO_VALIDATE" --filterExpression "DP < 5 " --filterName "LowCoverage" --filterExpression "QD < 1.5 " --filterName "LowQD" -- filterExpression "QUAL < 30.0" --filterName "VeryLowQual"--filterExpression "QUAL > 30.0 && QUAL < 50.0 " --filterName "LowQual" --clusterWindowSize 10-- cluster-size 3), after that we filtered callset at population level based on MAF>0.05 and missing rate<0.1, so the rare alleles showed low frequency. We plot all SNPs after hard filtration (shown below), the rare alleles exhibited similar pattern with Figure 4 in Nielsen et al., 2012. The two results have no conflict, so we would like to keep the original Fig.S3.

Well, the purpose of your figure is to show the SNPs you called is reasonable based on both folded and unfolded SFS. It would be better show the SFS without filtering based on MAF and missing rate. Otherwise, it is really misleading.

Minor:

I still feel confused about the why the authors need to filter the SNPs more than 3SNPS clustered in a 10-bp window. Please explain the rationale.

Response: We are working on a non-model organism for which there is no SNP data available. So following GATK best practice tutorial, we chose Hard Filtering instead of Variant Recalibration (VQSR) method to filter our variants callset (https://gatk.broadinstitute.org/hc/en-us/articles/360036434492-VariantFiltration). This parameter mark clusters of at least 3 SNPs that are within a window of 10 bases between them to avoid false positive sites. Please note that we selected these hard filtering values in attempting to optimize both high sensitivity and specificity together. We applied this analysis using the ‘clusterWindowSize10’ recommended by the GATK toolkit, 3 SNPs clustered in a 10-bp window is considered to be unreliable and would generate false positive effects, for more references see Bowen et al., 2011 and 2012, doi:10.1371/journal.pgen.1002050; doi.org/10.1534/genetics.111.136069.

Thanks for your explanation. It would be great that the authors can briefly add the rationale in the method part.
